# Input-specific deployment of the active zone scaffold RIM tunes synaptic release probability and plasticity

**DOI:** 10.1126/sciadv.aeh4857

**Published:** 2026-11-11

**Authors:** Rebecca Stark, Prapti Patel, Wanying Dong, Caleb Dehn, Dion Dickman

**Affiliations:** ^1^ Department of Neurobiology, University of Southern California, Los Angeles, CA USA.; ^2^ USC Neuroscience Graduate Program, University of Southern California, Los Angeles, CA USA.

## Abstract

The multidomain scaffold RIM (Rab3-interacting molecule) is widely viewed as a broadly required organizer of the presynaptic active zone, clustering voltage-gated Ca^2+^ (Ca_v_2) channels, priming synaptic vesicles, and supporting neurotransmitter release. Whether this reliance is uniform across synapses with distinct release probabilities, however, has been difficult to resolve at convergent inputs. Here, using neurotoxin-based silencing to isolate transmission at the *Drosophila* neuromuscular junction, we show that the requirement for RIM is highly input-specific: RIM is essential to empower transmission at a high–release probability phasic synapse, yet is largely dispensable at a neighboring low–release probability tonic synapse. This specificity extends to plasticity: RIM is required for acute homeostatic potentiation at the phasic input, but not for chronic potentiation at the tonic input. Super-resolution imaging reveals that RIM is positioned closer to Ca_v_2 channels at phasic synapses and, during acute plasticity, coordinates their nanoscale reorganization to drive the rapid expansion of the readily releasable pool.

## INTRODUCTION

Neural circuits rely on the precise routing of information through vast networks of synapses that exhibit notable diversity in structural and functional properties. A single postsynaptic neuron may receive thousands of convergent inputs, yet it must independently tune specific connections to distinct “set points” of synaptic strength, plasticity, and neuromodulatory sensitivity (*1*–*4*). This input-specific heterogeneity allows circuits to encode complex information, filtering high-frequency signals at some inputs while acting as high-fidelity relays at others. The presynaptic active zone (AZ) is a primary locus for this specialized transformation. Defined by a conserved nanostructure of protein scaffolds, the AZ controls neurotransmission by organizing voltage-gated Ca^2+^ (Ca_V_2) channels, delineating vesicle release sites, and positioning priming factors such as Unc13 to couple Ca^2+^ influx to vesicle fusion (*5*, *6*). While the molecular “parts list” of the AZ is largely conserved across species, how these components are differentially configured to generate the wide dynamic range of synaptic strength observed across diverse inputs, particularly at connections facing high activity demands, and how they are subsequently modified by plasticity remain poorly understood.

The multidomain scaffold RIM (Rab3-interacting molecule) is widely regarded as the central organizer of the AZ. RIM proteins contain multiple conserved interaction domains—including an N-terminal zinc finger flanked by α helices, a central PDZ domain, and C-terminal C2 domains separated by a proline-rich motif—that form a hub connecting virtually all other AZ components (*7*, *8*). Extensive biochemical and genetic studies in mammals and invertebrates have established RIM as a key component that anchors the release machinery: RIMs directly interact with Rab3 to recruit synaptic vesicles, bind Unc13/Munc13 via their N-terminal zinc finger to drive vesicle priming, and physically tether Ca_V_2 channels to the AZ via interactions with RIM-binding protein (RBP) and a direct PDZ domain-channel C terminus interaction (*7*–*14*). These functions are executed by distinct, modular domains: The zinc finger autonomously activates priming by disrupting autoinhibitory Munc13 homodimerization, while a separate PDZ-RBP module mediates Ca^2+^ channel tethering (*7*, *11*, *12*). Through this multivalent architecture, RIM simultaneously organizes the three core prerequisites for fast neurotransmitter release: a primed vesicle pool, clustered Ca^2+^ channels, and tight spatial coupling between them, consistent with the prevailing view of RIM as a central organizer of release machinery at synapses.

Consequently, the loss of RIM generally leads to a reduction in Ca^2+^ channel density and impaired synaptic vesicle fusion (*7*, *15*, *16*). However, whether RIM contributes uniformly across functionally distinct synapses is increasingly questioned. Emerging evidence suggests that RIM requirements may vary substantially between synapse subtypes—for instance, between excitatory and inhibitory synapses in the hippocampus, or in the differential regulation of dense-core vesicle release at neuromodulatory terminals (*10*, *12*, *17*–*21*). These findings suggest that rather than serving as a static “brick” in the AZ wall, RIM may function as a tunable variable, differentially deployed to match the specific performance requirements of a synapse. However, a rigorous mechanistic dissection of this specialization has been hindered by the difficulty of assessing RIM function at distinct, convergent inputs within an intact circuit and by the genetic redundancy of *rim* genes in mammals.

The *Drosophila* neuromuscular junction (NMJ) offers a powerful genetic system to resolve these questions about input-specific RIM functions. At the fly NMJ, a single *rim* gene encodes the conserved functions of stabilizing Ca_V_2 channels and recruiting the readily releasable pool (RRP) (*15*). Notably, the fly genome also encodes *fife*, a Piccolo-RIM–related AZ scaffold that promotes neurotransmitter release at this NMJ (*22*, *23*), and RIM operates within a partially redundant scaffold that includes RBP and ELKS/BRP (*9*, *10*, *24*, *25*). Furthermore, beyond basal transmission, subsequent work identified RIM as a critical effector of presynaptic homeostatic potentiation (PHP), a form of adaptive plasticity that up-regulates presynaptic neurotransmitter release following the genetic loss or pharmacological blockade of postsynaptic receptors (*26*). Specifically, RIM was reported to mediate the homeostatic expansion of the RRP required to restore synaptic strength (*26*). However, a critical confound exists in these foundational studies: Electrophysiological recordings were performed on muscles co-innervated by two distinct motor neurons—the tonic “Type Ib” (MN-Ib) and the phasic “Type Is” (MN-Is)—which differ profoundly in their basal release properties, short-term plasticity, and AZ nanoarchitecture (*27*–*32*). Notably, although MN-Ib exhibits a lower initial release probability than MN-Is, its strong short-term facilitation can bring cumulative release during trains closer to that of the depressing MN-Is input; the two inputs therefore differ principally in initial release probability and short-term dynamics rather than in total output (*29*). Furthermore, previous imaging characterizing *rim* mutants focused predominantly on MN-Ib terminals, leaving potential differences at MN-Is unexamined and introducing a disconnect between blended electrophysiological phenotypes and input-restricted structural analyses. Because standard electrophysiological recordings represent an ambiguous composite of these dramatically divergent inputs, it has remained unknown whether RIM is universally required for basal transmission and plasticity at both connections or whether the phenotypes observed in *rim* mutants reflect the selective failure of a single synaptic subtype.

Here, we use botulinum neurotoxin–based genetic silencing to isolate transmission from tonic MN-Ib and phasic MN-Is inputs and rigorously test the synapse-type specificity of RIM. We find that, across these two inputs, the requirement for RIM is highly input-specific: While RIM is essential for high release probability emission at phasic MN-Is synapses, basal transmission at tonic MN-Ib is largely unchanged in *rim* null mutants. This input specificity extends into plasticity, where RIM is required for the acute expression of PHP at MN-Is but is dispensable for the chronic maintenance of PHP at MN-Ib. Mechanistically, endogenous tagging and super-resolution imaging reveal that RIM is differentially organized at the nanoscale, positioned closer to Ca_V_2 clusters at MN-Is where it maintains tight Ca_V_2-vesicle coupling and supports rapid RRP expansion. Furthermore, during acute plasticity, RIM coordinates the homeostatic reorganization of Ca_V_2 channel clusters to recruit additional vesicles for fusion. These findings indicate that, across these two convergent inputs, reliance on the *rim* gene is not uniform; rather, it is differentially deployed as a tunable module that boosts synaptic strength and plasticity at a high-release-probability connection.

## RESULTS

### RIM selectively promotes neurotransmitter release at phasic MN-Is synapses

To determine whether the AZ scaffold RIM contributes uniformly to synaptic transmission, we exploited the *Drosophila* larval NMJ, where the tonic MN-Ib and phasic MN-Is co-innervate muscle 6, exhibiting dramatically distinct release properties (Fig. 1, A and B). Specifically, tonic MN-Ib terminals exhibit lower release probability and facilitate upon repeated stimulation, while phasic MN-Is terminals have a higher *P*_r_ and depress during repeated stimulation (*29*, *33*, *34*). We generated targeted *rim* null mutant alleles in an isozygous genetic background (*w^1118^*), ensuring consistency across all genotypes evaluated in this study. Using CRISPR-Cas9 gene editing, we isolated two alleles (*rim^RS1^* and *rim^RS2^*) (fig. S2A), confirming the expected anatomic and electrophysiologic phenotypes reported previously (fig. S2, B to F) (*15*, *26*). These phenotypes included normal miniature events and an ∼50% reduction in evoked responses during blended, co-stimulated (Is+Ib) transmission at NMJs (Fig. 1, C to E, and fig. S2, B to F). Given that both *rim* alleles exhibited similar expected phenotypes, we focused exclusively on *rim^RS1^* for the remainder of our experiments.

**Fig. 1.**
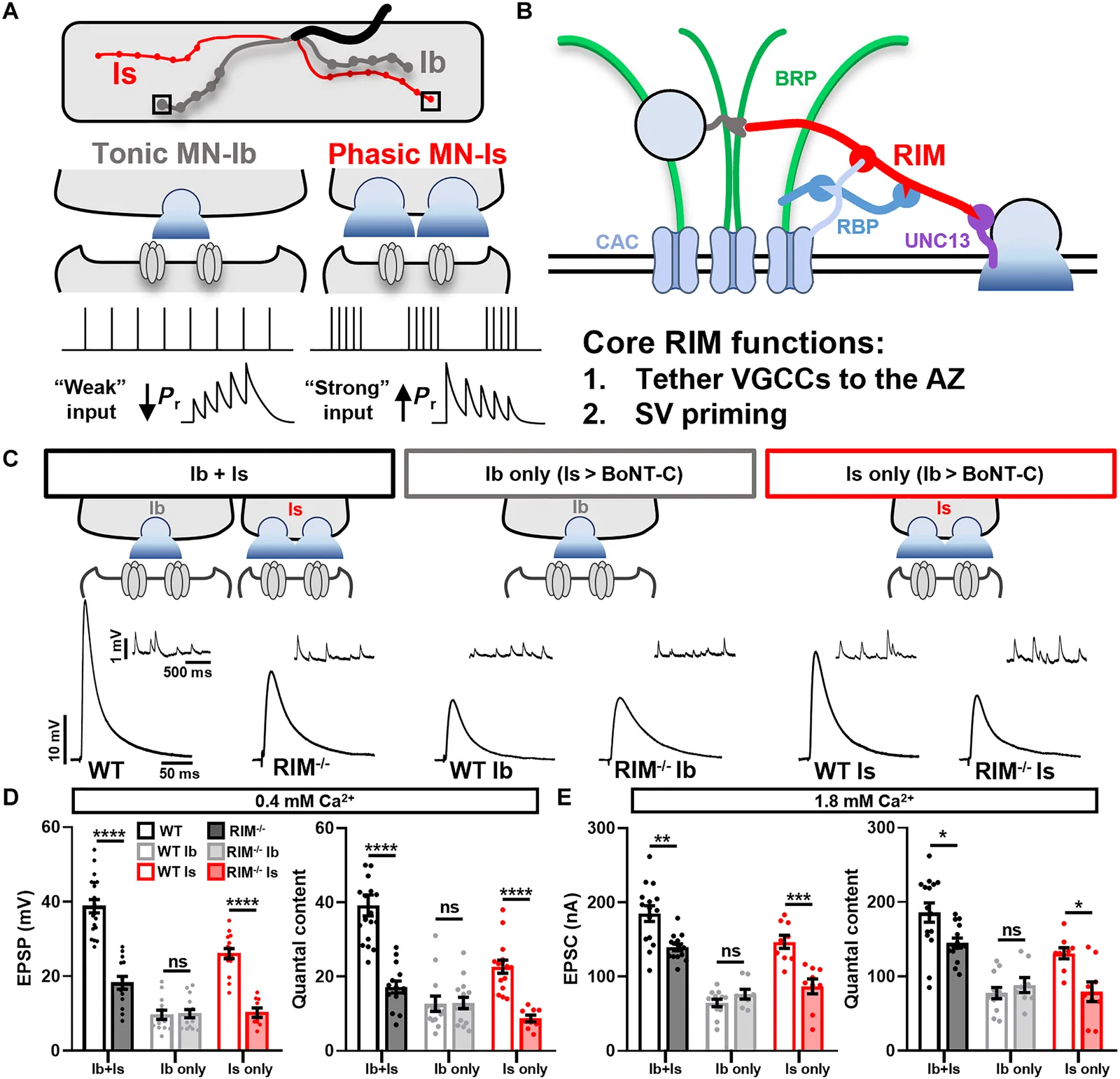
RIM selectively promotes neurotransmitter release at phasic MN-Is synapses. (**A**) Schematic of the *Drosophila* NMJ, where motor neurons Ib and Is co-innervate abdominal muscle 6. Tonic MN-Ib synapses function with low release probability, while phasic MN-Is synapses operate with high release probability. (**B**) Schematic of the AZ, highlighting key interactions of RIM with associated release machinery; RIM is drawn to illustrate these interactions, and its full domain architecture (zinc finger–PDZ–C2A–C2B) is shown in fig. S2A. (**C**) Representative mEPSP and EPSP traces at baseline (wild type; *w^1118^*) and in *rim* mutants (*w*;;*rim^RS1^*) during co-stimulation (Ib + Is) or following input-specific silencing with BoNT-C (MN-Ib or MN-Is only, achieved by BoNT-C expression in the convergent input). Evoked amplitude is unchanged at MN-Ib in the absence of *rim*, while transmission from MN-Is is reduced by ∼60%. (**D** and **E**) Quantification of evoked EPSP amplitude and quantal content values at 0.4 mM (D) and 1.8 mM (E) extracellular [Ca^2+^], revealing selective deficits exclusively at *rim* MN-Is. Genotypes are indicated by bar fill as shown in the panel key (WT, open; *rim*^*−/−*^, filled; Ib, gray; Is, red). Error bars indicate ±SEM. *n* values and full statistical details are provided in table S1.

To resolve the input-specific requirements of *rim* at tonic versus phasic synapses, we isolated MN-Ib or MN-Is transmission via the targeted expression of botulinum neurotoxin C (BoNT-C), which silences transmission at the convergent input (i.e., MN-Is > BoNT-C to isolate MN-Ib, referred to as “Ib only,” and vice versa for “Is only”) (*29*, *35*). At isolated MN-Ib synapses in *rim* mutants, we observed no significant change in excitatory postsynaptic potential (EPSP) responses in *rim* mutants compared to wild-type controls at reduced extracellular Ca^2+^ (0.4 mM) (Fig. 1, C and D), nor did EPSC amplitudes differ under higher Ca^2+^ conditions (Fig. 1E and fig. S1, A and B). We probed these release properties in further detail at isolated MN-Ib synapses, finding no significant differences in EPSC amplitude or quantal content across a wide range of extracellular Ca^2+^ conditions (0.4 to 6 mM). Furthermore, failure analysis and paired-pulse facilitation revealed no differences in release probability for *rim* mutants at MN-Ib (fig. S1, A to H). These findings suggested that all observed defects in neurotransmission following the loss of *rim* derive exclusively from alterations in phasic MN-Is release properties.

At isolated MN-Is synapses, the loss of *rim* reduced release by a sufficient magnitude to explain the defects previously reported, which were formerly interpreted as resulting from combined MN-Ib and MN-Is decrements. Specifically, both EPSP and EPSC amplitudes were reduced across all extracellular Ca^2+^ concentrations evaluated in *rim* MN-Is synapses (Fig. 1, C to E, and fig. S1, C and D), while failure and paired-pulse analyses confirmed a significantly decreased *P*_r_ (fig. S1, E to H). Together, these data demonstrate that *rim* is selectively required to support high release probability transmission at phasic MN-Is synapses, whereas tonic MN-Ib transmission remains essentially unperturbed in its absence.

### RIM is enriched at MN-Is AZs

We next asked whether differences in RIM abundance or nanoscale organization might explain its apparent importance in promoting neurotransmitter release selectively at phasic MN-Is synapses. To address this, we generated an endogenously tagged *rim* allele using a short ALFA tag (*rim^ALFA^*), targeting a region near the N-terminal Zinc Finger domain previously validated in a recent study (*36*, *37*) (fig. S2A). Characterization of *rim^ALFA^* larvae confirmed normal synaptic growth, transmission, and PHP plasticity expression compared to controls (fig. S2, G to K), validating the fact that RIM was unperturbed by the tag and justifying its use for localization analyses.

We first systematically examined six central AZ components across MN-Ib and -Is terminals to determine whether RIM or any other proteins exhibited differential localization or abundance. Confocal imaging of RIM^ALFA^ alongside BRP, RBP, CAC (*cac^sfGFP^*), Unc13A, and Unc13B revealed no significant differences in CAC, Unc13A, or Unc13B levels across the two input types (Fig. 2, A and C). Additionally, we confirmed previously reported reductions in intensity for BRP and RBP at Is synapses relative to Ib (Fig. 2, A and B) (*29*, *32*). However, RIM intensity was significantly higher at MN-Is AZ compared to MN-Ib (Fig. 2, A and B), which is notable as it represents the only AZ component examined that enriched at Is over Ib synapses. Next, we used super-resolution (stimulated emission depletion; STED) microscopy to resolve the nanoscale organization of these components. Within a single AZ labeled by the “T-bar” scaffold BRP, we observed approximately two RIM puncta at both MN-Ib and MN-Is, indicating no significant difference in cluster number (Fig. 2, D and E). However, when we imaged RIM and Ca_V_2 (CAC) channels, we observed significantly tighter distances between RIM and CAC at MN-Is compared to MN-Ib (Fig. 2, F and G). Thus, RIM is not only enriched but also positioned closer to Ca_V_2 nanodomains at phasic MN-Is synapses.

**Fig. 2.**
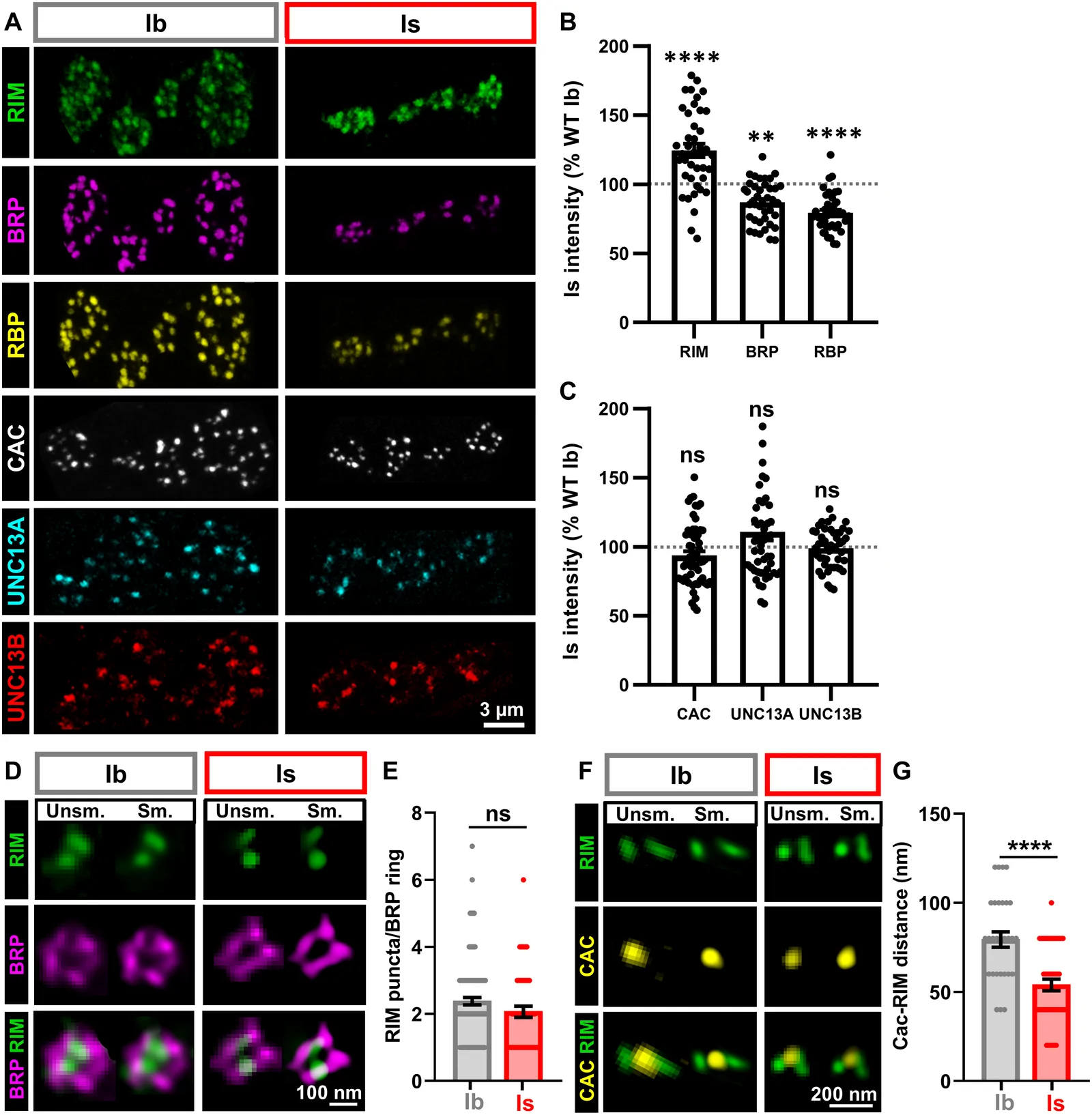
RIM is uniquely abundant at MN-Is AZs and localizes close to Ca_V_2 channels. (**A**) Representative confocal images of muscle 6 NMJs immunostained for RIM (ALFA), BRP, RBP, and CAC (GFP) at MN-Ib and -Is. BRP, RIM, and RBP were co-immunostained and quantified together. CAC and Unc13A/Unc13B were each imaged in separate sample sets with BRP included only as an AZ marker and not quantified. (**B**) Quantification of mean immunofluorescence intensity for the indicated antigens at MN-Is, normalized to the within-session wild-type MN-Ib mean (% MN-Ib). RIM intensity is uniquely higher at MN-Is, whereas BRP and RBP levels are higher at MN-Ib. (**C**) Quantification as in (B) for CAC, Unc13A, and Unc13B, showing similar intensities between MN-Ib and -Is. (**D**) Representative STED images of single AZs immunostained for BRP and RIM (ALFA) at MN-Ib and MN-Is. (**E**) Quantification of RIM puncta per BRP ring, showing no difference between inputs. (**F**) Representative STED images of single AZs immunostained for CAC (GFP) and RIM (ALFA). For representative STED images in (D) and (F), each image is shown as an unsmoothed rendering of the deconvolved data (Unsm., left) alongside a bicubic-interpolated rendering for display clarity only (Sm., right); only linear brightness/contrast adjustments were applied. (**G**) Quantification of CAC-RIM distance, which is significantly shorter at MN-Is than MN-Ib. STED distances reflect the following detected epitopes: RIM, N-terminal ALFA tag (zinc finger proximal); CAC, N-terminal sfGFP knock-in (*cac^sfGFP-N^*); BRP C terminus, the nc82 epitope. Absolute interprotein distances should be interpreted in light of these epitope positions and antibody offset (∼10 to 15 nm). All STED measurements were restricted to planar, en-face AZs (see Materials and Methods). Error bars indicate ±SEM. *n* values and full statistical details are shown in table S1.

We next sought to determine how AZ organization at MN-Ib and MN-Is is affected by loss of *rim*, given that RIM is a conserved AZ scaffold implicated in vesicle priming and Ca^2+^ channel organization at synapses (*7*, *13*, *15*). We systematically assessed the remaining five AZ components (BRP, RBP, CAC, Unc13A, and Unc13B) at each input using confocal microscopy. Consistent with previous studies showing that RIM contributes to Ca^2+^ channel recruitment and release-site organization at the *Drosophila* NMJ (*15*, *26*), we observed a small but expected reduction in CAC levels, while BRP and RBP levels were unchanged at both inputs (Fig. 3, A, B, E, and F).

**Fig. 3.**
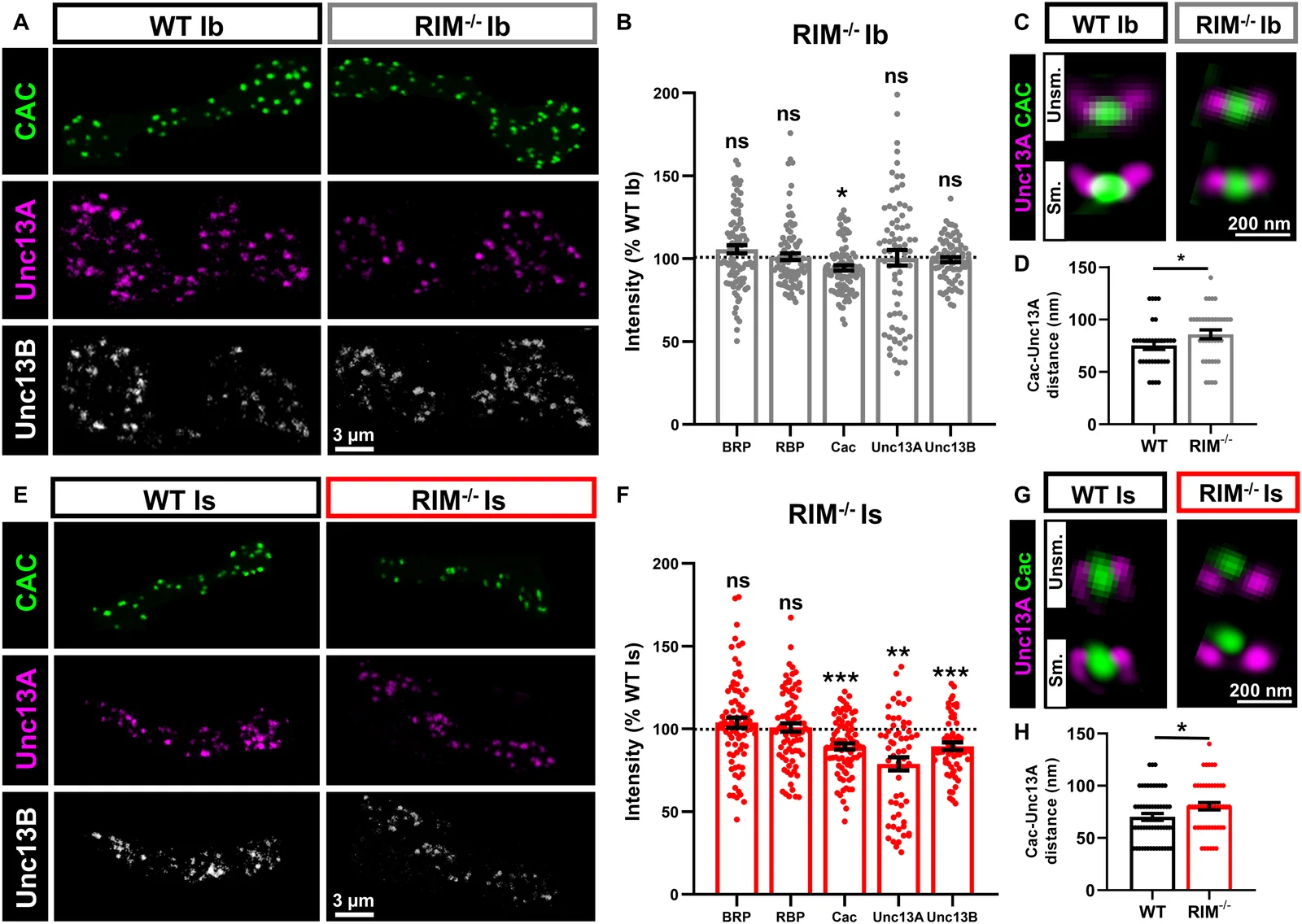
RIM supports the accumulation of CAC and positioning of Unc13A near Ca_V_2 channels. (**A**) Representative confocal images of MN-Ib NMJs immunostained for CAC, Unc13A, and Unc13B in wild type and *rim* mutants. (**B**) Quantification of mean immunofluorescence intensity for the indicated antigens in *rim* mutants as a percentage of wild type. Loss of *rim* leads to a small, selective decrease in CAC levels. (**C**) Representative STED images of single AZs immunostained for Unc13A and CAC at MN-Ib in wild type and *rim* mutants. For representative STED images in (C) and (G), each image is shown as an unsmoothed rendering of the deconvolved data (Unsm., top) alongside a bicubic-interpolated rendering for display clarity only (Sm., bottom); only linear brightness/contrast adjustments were applied. (**D**) Quantification of Cac-Unc13A distances at MN-Ib NMJs, with moderate but significant increase in CAC-Unc13A distance in the absence of *rim*. (**E** to **H**) Corresponding data for MN-Is. At MN-Is, loss of *rim* significantly reduces CAC, Unc13A, and Unc13B levels and causes a small but significant increase in Cac-Unc13A distance. Error bars indicate ±SEM. *n* values and full statistical details are shown in table S1.

We next examined the vesicle-priming factors Unc13A and Unc13B, which occupy distinct positions at release sites and contribute to synaptic vesicle–Ca^2+^ channel coupling at the NMJ (*38*). While neither Unc13 isoform changed at MN-Ib synapses, loss of *rim* reduced both Unc13A and Unc13B (to ∼74 and ∼89% of wild type, respectively) specifically at MN-Is terminals (Fig. 3, A, B, E, and F). Unc13A intensity at MN-Is varied considerably across boutons, likely reflecting bouton-to-bouton heterogeneity in active-zone number and maturation together with the differential release-site positioning of the two isoforms noted above (*38*). To confirm that the reduction was robust to this spread, we performed an additional genotype-blinded acquisition and analysis in which files were coded prior to quantification; this reproduced the significant reduction of Unc13A in *rim* mutants at MN-Is (Materials and Methods).

Given the pronounced reduction of Unc13A selectively at MN-Is AZs, we next asked whether the spatial relationship between Unc13A and Ca^2+^ channels was altered in the absence of RIM. Using STED imaging, which has previously revealed the nanoscale organization of Unc13 isoforms relative to Ca^2+^ channels at *Drosophila* AZs (*38*), we measured CAC-Unc13A distances in *rim* mutants. In the absence of *rim*, Unc13A was positioned modestly but substantially farther from CAC at both MN-Is and MN-Ib synapses (Fig. 3, C, D, G, and H), indicating that RIM helps stabilize Unc13A near Ca_V_2 channels at both inputs. Notably, this looser Unc13A-CaV2 coupling was accompanied by a pronounced loss of Unc13A abundance only at MN-Is, whereas MN-Ib retained normal Unc13A levels (Fig. 3, E and F). Thus, loss of *rim* compromises both the amount and the channel proximity of the dominant priming factor at phasic MN-Is, but affects only its positioning at tonic MN-Ib. Because Unc13A is displaced from Ca_v_2 at both inputs yet depleted only at MN-Is, it is the input-specific loss of Unc13A abundance, rather than its repositioning, that parallels the selective functional requirement for RIM at MN-Is.

### RIM selectively promotes PHP

We next focused on clarifying whether the unanticipated, input-specific functions of *rim* at phasic MN-Is synapses extend to roles in plasticity as well. PHP is a well-studied form of plasticity modeled at the *Drosophila* NMJ, where pharmacological blockade or genetic loss of postsynaptic glutamate receptors triggers a retrograde, adaptive enhancement in presynaptic release to maintain stable synaptic strength (*39*–*42*). A previous study identified *rim* as essential for acute PHP (*26*), which was interpreted as resulting from a genetically separable role in Ca^2+^ influx and the expansion of the RRP. Because the blended physiological responses from MN-Ib and -Is confounded this interpretation, an alternative hypothesis is that the loss of *rim* did not actually illuminate genetically separable processes in PHP, but rather that these processes are dictated by input-specific functions during PHP. A recent study demonstrated that two forms of PHP use distinct mechanisms, and target distinct inputs, to achieve stable NMJ strength (*28*). “Chronic PHP,” induced through the genetic loss of the *GluRIIA* subunit, selectively drives enhanced presynaptic Ca^2+^ influx at MN-Ib to promote enhanced vesicle release and maintain stable synaptic strength (schematized in Fig. 4A). In contrast, “acute PHP,” induced through the pharmacological application of the glutamate receptor antagonist philanthotoxin (PhTx), selectively targets MN-Is for homeostatic modulation, mobilizing expanded RRP pools to drive the increased transmitter release that stabilizes synaptic strength (Fig. 4E). Given these critical insights into the input-specific mechanisms orchestrating PHP, we focused on accurately assessing *rim*’s functions across these distinct forms of plasticity.

**Fig. 4.**
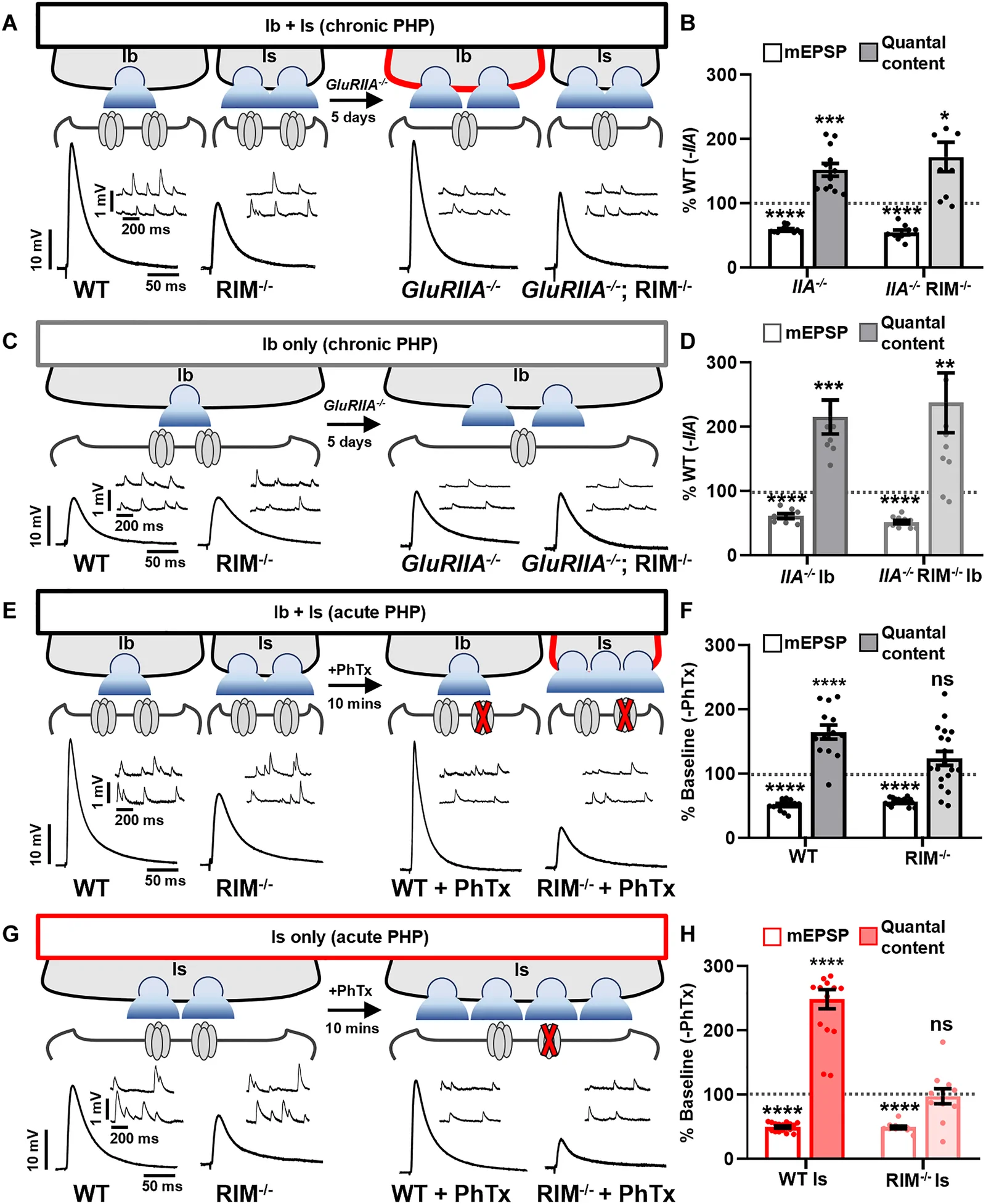
RIM selectively promotes acute PHP at MN-Is. (**A**) Representative EPSP traces for baseline (WT: *w^1118^*), chronic PHP (*GluRIIA*^*−/−*^: *w*;*GluRIIA^PV3^*), *rim^−/−^* (*w*;;*rim^−/−^*), and chronic PHP in *rim ^−/−^* (*w*;*GluRIIA^PV3^*;*rim ^−/−^*) NMJs at 0.4 mM extracellular [Ca^2+^]. (**B**) Quantification of mEPSP amplitude and quantal content normalized as a percentage of baseline. Quantal content is significantly enhanced in both WT and *rim^−/−^*, indicating robust chronic PHP expression. (**C**) Representative EPSP traces for isolated MN-Ib following selective expression of BoNT-C in MN-Is. (**D**) Quantification of the data shown in (C), confirming chronic PHP is expressed at MN-Ib lacking *rim*. (**E** and **F**) Corresponding data for acute PHP (PhTx application) at co-stimulated inputs (Ib + Is). Quantal content does not change in *rim* + PhTx, indicating a block in acute PHP expression. (**G** and **H**) Corresponding data for acute PHP at isolated MN-Is. Acute PHP is not expressed in isolated *rim* MN-Is NMJs. Error bars indicate ±SEM. *n* values and full statistical details are shown in table S1.

Whether *rim* is necessary for chronic PHP has previously remained unknown. We therefore generated *GluRIIA*;*rim* double mutants to assess chronic PHP using blended (Ib + Is) physiology. We found that while presynaptic release (quantal content) was robustly enhanced in *GluRIIA* mutants alone, a similar enhancement was observed in *GluRIIA*;*rim* double mutants, demonstrating that *rim* is not required for chronic PHP expression (Fig. 4, A and B). As expected, in isolated MN-Ib recordings using BoNT-C expression at MN-Is, chronic PHP was robustly expressed despite the loss of *rim* (Fig. 4, C and D). In contrast, we confirmed that *rim* is required for acute PHP expression when evaluating co-stimulated inputs (Fig. 4, E and F), as previously reported (*26*). Crucially, PhTx application to isolated *rim* mutant MN-Is terminals revealed that *rim* is strictly necessary at phasic MN-Is for acute PHP expression (Fig. 4, G and H). Together, these data demonstrate that *rim*’s previously assigned functions in Ca^2+^ channel stabilization and RRP modulation do not genetically separate PHP mechanisms but rather constitute input-specific functions at high *P*_r_ phasic MN-Is synapses that extend to homeostatic plasticity, proving that *rim* is selectively required for acute PHP at these phasic inputs.

Because acute PHP selectively targets phasic MN-Is synapses by enhancing the RRP (*28*), we next determined whether *rim* was necessary for this process. To estimate the RRP, we stimulated NMJs at high frequency (60 Hz) under elevated *P*_r_ conditions (3 mM extracellular Ca^2+^) and plotted the cumulative EPSC; a linear fit from stimulus 19 to 30 was back-extrapolated to time 0 and divided by the cell’s mean miniature excitatory postsynaptic potential (mEPSP) amplitude to estimate the pool size (Fig. 5, A and B) (*43*). This analysis revealed that the baseline RRP was reduced by ∼50% at MN-Is in *rim* mutants under these elevated-Ca^2+^ (3 mM) conditions (Fig. 5, A to C) but remained unaffected at MN-Ib (table S1). Following acute PHP induction by PhTx, control MN-Is exhibited a robust enhancement of RRP size, while in *rim* mutants, the RRP failed to increase (Fig. 5, A to C), consistent with—and now resolved to the MN-Is input—the previously published finding from combined MN-Ib and -Is recordings (*26*). This homeostatic defect was not an artifact of elevated Ca^2+^: At physiological 1.5 mM Ca^2+^, PhTx expanded the RRP ∼2.6-fold in controls but failed to do so in *rim* mutants, even though baseline RRP was comparable between genotypes under these conditions (table S1). The steady-state vesicle recruitment rate was also reduced in *rim *mutants** at MN-Is (table S1), consistent with a broader role for RIM in vesicle replenishment. Together, these results indicate that *rim* is required for the homeostatic expansion of the RRP and promotes steady-state vesicle recruitment, specifically at high-release-probability phasic MN-Is synapses.

**Fig. 5.**
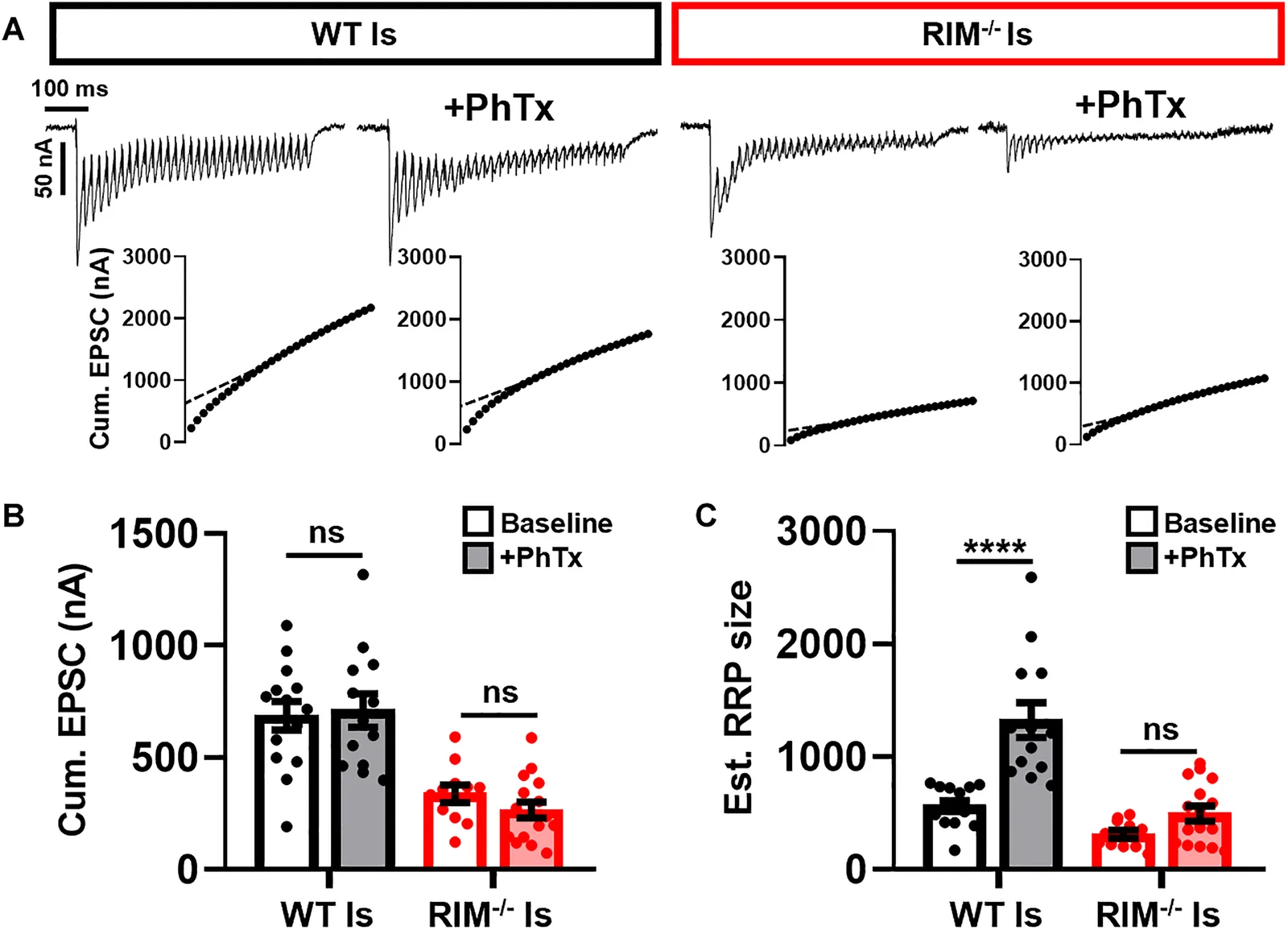
RIM is required for the homeostatic increase in the RRP that enables acute PHP. (**A**) Representative traces of 30 EPSCs from wild type and *rim* mutants at isolated MN-Is NMJs stimulated at 60 Hz in 3 mM extracellular [Ca^2+^], at baseline and after PhTx application. Bottom: Averaged cumulative EPSC amplitudes (running sum of evoked EPSC amplitudes across the 30-stimulus train). A linear fit to stimuli 19 to 30 was back-extrapolated to time 0, and the *y* intercept was divided by the mean mEPSP amplitude to estimate RRP size; the slope of this fit approximates steady-state vesicle recruitment rate. Three millimolar [Ca^2+^] was used to drive near-complete depletion of the releasable pool required for reliable back-extrapolation, which is not achieved at physiological [Ca^2+^]. (**B** and **C**) Quantification of cumulative EPSC (B) and estimated RRP size (C). At baseline, RRP size is reduced ∼50% in *rim* mutants at MN-Is relative to wild type. Following acute PHP induction by PhTx, wild-type MN-Is synapses exhibit a robust homeostatic increase in RRP size that is absent in *rim* mutants. Equivalent measurements at physiological 1.5 mM [Ca^2+^], together with steady-state vesicle recruitment rates (slope of the fit to stimuli 19 to 30) for all conditions, are reported in table S1. Error bars indicate ±SEM. *n* values and full statistical details are shown in table S1.

### RIM coordinates the homeostatic reorganization of Ca_v_2 nanostructure

Last, we examined RIM in the homeostatic reorganization of AZ nanostructure during acute PHP. AZ components at MN-Ib and -Is remodel following both chronic and acute PHP (*44*–*48*): At MN-Is, BRP, RBP, and CAC increase in intensity after PhTx application (*28*, *32*), and previous super-resolution imaging has described a “compaction” of AZ nanostructure (*28*, *36*, *49*), including compression of CAC into a smaller area at AZs (*28*, *49*). Because STED reports the apparent area of epitope-defined clusters rather than molecular density, we describe our measurements as changes in apparent CAC cluster area or nanoscale reorganization rather than compaction. A reduction in apparent area cannot by itself distinguish increased molecular packing from lateral redistribution, altered molecular abundance, or differences in epitope accessibility (see Discussion). RIM itself responded to acute PHP, increasing in fluorescence intensity at MN-Is AZs following PhTx (Fig. 6, A and B). By STED, the number of RIM puncta per AZ was unchanged (Fig. 6, C and D), while the RIM-CAC distance decreased from ∼60 to ∼40 nm (Fig. 6, E and F), reflecting closer RIM-Ca_v_2 apposition during acute PHP.

**Fig. 6.**
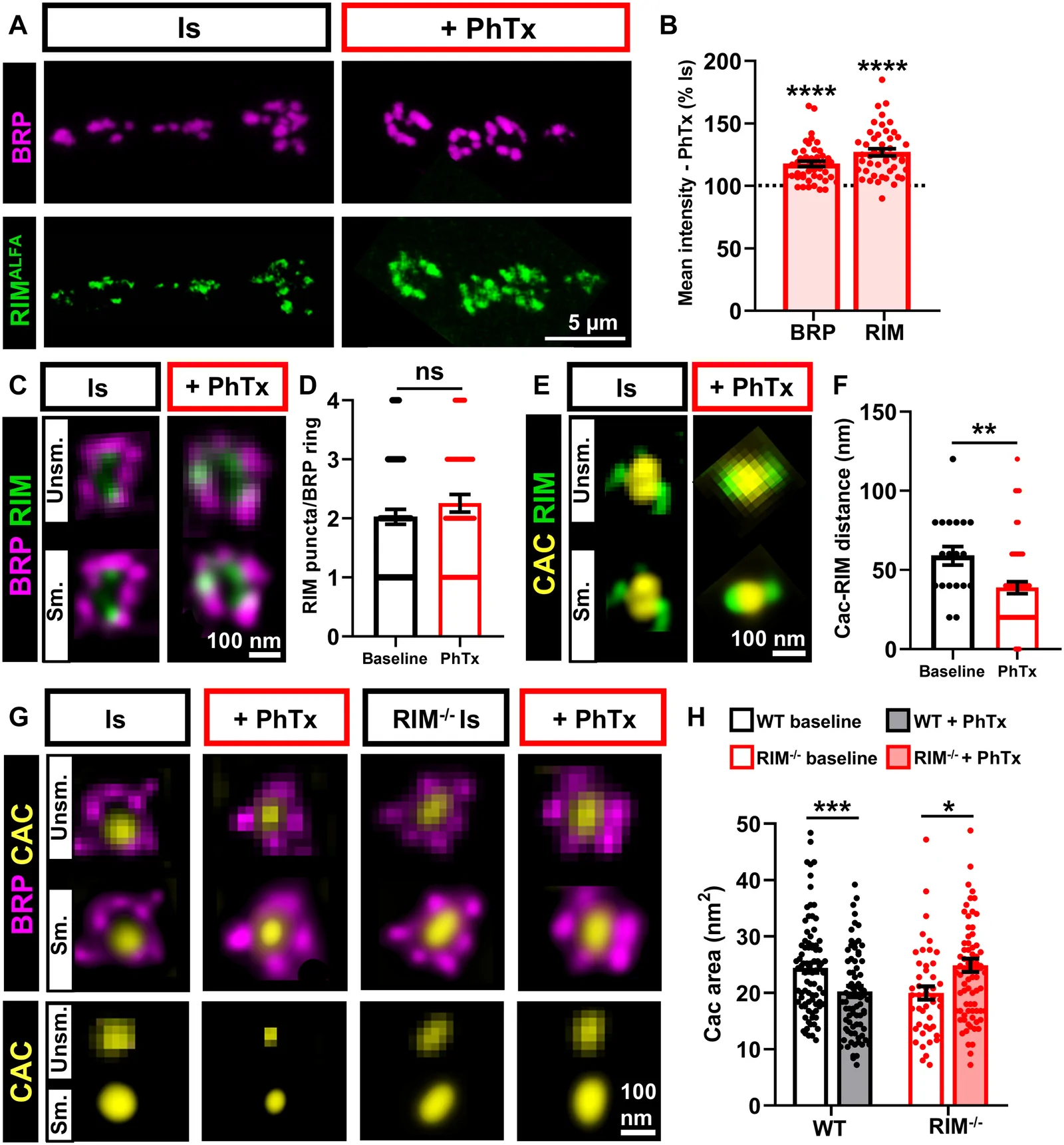
RIM is positioned closer to Ca_V_2 channels and coordinates their nanoscale reorganization during acute PHP. (**A**) Representative confocal images of MN-Is NMJs immunostained for BRP and RIM (ALFA) at baseline and after PhTx application. (**B**) Quantification of BRP and RIM intensities after PhTx application as a percentage of baseline, indicating enhanced intensity of both during acute PHP. (**C**) Representative STED images of single AZs immunostained for RIM (Rim^ALFA^) and BRP or CAC (GFP) in the indicated conditions. For representative STED images in (C), (E), and (G), each image is shown as an unsmoothed rendering of the deconvolved data (Unsm., top) alongside a bicubic-interpolated rendering for display clarity only (Sm., bottom); only linear brightness/contrast adjustments were applied. (**D**) Quantification of RIM puncta per BRP ring, indicating that the number of RIM puncta does not change during acute PHP. (**E**) Quantification of distance between CAC and RIM puncta at baseline and after PhTx application, showing tighter coupling during acute PHP. (**F**) Representative STED images of single AZs immunostained for BRP and CAC at MN-Is at baseline and after PhTx application in wild type and *rim* mutants. (**G**) Quantification of CAC area after PhTx application as a percentage of baseline. Apparent CAC cluster area is reduced during acute PHP in wild type but not in *rim* mutants. Error bars indicate ±SEM. *n* values and full statistical details are provided in table S1.

We next asked how CAC nanostructure responds to acute PHP in wild-type versus *rim* mutant MN-Is AZs. In wild type, PhTx reduced the apparent area of CAC clusters (Fig. 6, G and H). In *rim* mutants, baseline CAC cluster area was already reduced relative to wild type—consistent with less CAC at AZs, although we cannot exclude altered cluster organization—and, following PhTx, CAC area increased rather than decreased, opposite to the wild-type response. We favor a model in which CAC accumulates at *rim* mutant AZs during acute PHP but does not undergo the reduction in apparent cluster area observed in wild type, although alternative explanations for the area increase remain to be tested. Together, these data indicate that the homeostatic reduction in CAC cluster area that characterizes acute PHP at wild-type MN-Is AZs is RIM-dependent.

What adaptive functions would the RIM-dependent reduction in CAC cluster area serve to promote enhanced neurotransmitter release following acute PHP signaling? One attractive possibility is that the smaller area of CAC channels and associated machinery enables more synaptic vesicles to be located near Ca^2+^ sources to be released during evoked activity. To test this hypothesis, we subjected isolated wild type and *rim* mutant MN-Is synapses to EGTA-AM incubation at baseline and following PhTx application (Fig. 7A). EGTA-AM chelates Ca^2+^ diffusing from Ca_V_2 channels before it can reach “loosely coupled” synaptic vesicles located farther from the source (*50*, *51*). At baseline, EGTA-AM had little effect on wild-type MN-Is transmission, indicating tight vesicle-channel coupling, whereas it markedly reduced release at *rim* mutant synapses (Fig. 7, A and B). This greater EGTA sensitivity reflects an increased proportion of loosely coupled vesicles in *rim* mutants, consistent with RIM positioning Unc13A near CAC channels to drive tightly coupled fusion (Fig. 3, G and H). Following PhTx, EGTA-AM produced a further reduction in wild-type release that was absent in *rim* mutants (Fig. 7, A and B). Thus, these results suggest that acute PHP recruits additional loosely coupled vesicles for release through a RIM-dependent mechanism. STED imaging revealed that acute PHP reduces the distance between CAC and Unc13A, and that this enhanced proximity is RIM-dependent (Fig. 7, C and D). Together, these results support a model in which RIM homeostatically reorganizes AZ nanostructure during acute PHP, reducing the apparent area of Ca_v_2 clusters and positioning Unc13A, which defines sites for synaptic vesicle fusion, near Ca^2+^ sources to recruit additional loosely coupled vesicles. We present a schematic summarizing these roles for RIM in baseline and homeostatic plasticity specifically at phasic, high-release-probability synapses (Fig. 7E).

**Fig. 7.**
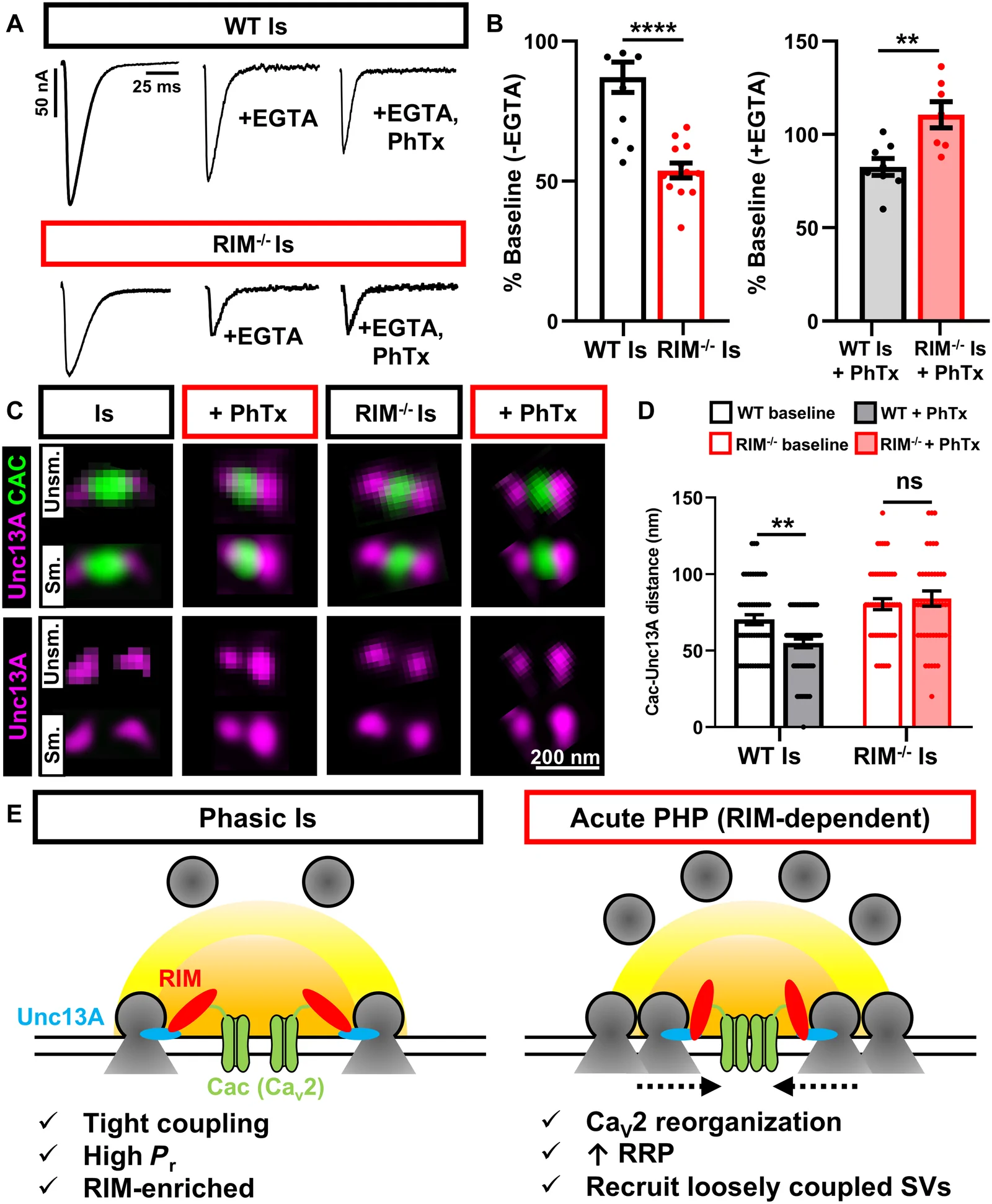
RIM is required for the enhanced Ca^2+^ coupling in acute PHP. (**A**) Representative EPSC traces at 1.8 mM [Ca^2+^] from isolated wild-type or *rim* MN-Is synapses at baseline, following EGTA-AM application, and following EGTA-AM with PhTx. (**B**) Quantification of EPSC amplitude after EGTA treatment normalized to baseline (left) or after PhTx application (right). A higher proportion of vesicles are loosely coupled in *rim* mutants at baseline. Following PhTx application, loosely coupled vesicles homeostatically increase in wild-type synapses, but remain unchanged in *rim* mutants. (**C**) Representative STED images of single MN-Is AZs immunostained for CAC and Unc13A at baseline and after PhTx application. For representative STED images in (C), each image is shown as an unsmoothed rendering of the deconvolved data (Unsm., top) alongside a bicubic-interpolated rendering for display clarity only (Sm., bottom); only linear brightness/contrast adjustments were applied. (**D**) Quantification of CAC-Unc13A distance, showing that acute PHP drives a RIM-dependent tightening of CAC-Unc13A nano-organization. (**E**) Schematic illustrating the role of RIM in promoting neurotransmission selectively at phasic MN-Is synapses at baseline and during acute PHP plasticity. Error bars indicate ±SEM. *n* values and full statistical details are provided in table S1.

## DISCUSSION

The AZ scaffold RIM is widely regarded as a central, broadly required organizer of the presynaptic release machinery. Using neurotoxin-based silencing to isolate transmission at convergent inputs of the *Drosophila* NMJ, we find that reliance on the *rim* gene instead varies with the release strategy of a given input: At the two inputs examined here, the tonic, low-*P*_r_ MN-Ib synapse functions remarkably well in the absence of *rim*, whereas high-*P*_r_ phasic MN-Is transmission depends critically on it. This input specificity concerns the *rim* gene at these two inputs and does not imply that presynaptic scaffolding is globally dispensable at MN-Ib, where redundant scaffolds are likely to sustain release (see below). That the same silencing manipulation leaves MN-Ib largely unaffected yet strongly impairs MN-Is argues against any nonspecific consequence of BoNT-C silencing, consistent with prior evidence that input silencing at this NMJ does not induce heterosynaptic changes (*35*). More broadly, these findings add to a growing appreciation of the molecular diversity of AZs (*1*, *3*, *4*), indicating that even a core scaffold can be differentially deployed to shape the functional properties of distinct synaptic connections.

To understand why RIM is selectively essential at phasic MN-Is terminals, we considered the distinct release strategies of these convergent inputs. MN-Ib and -Is are developmentally related glutamatergic motor neurons that express largely overlapping molecular machinery (*31*, *52*), yet they achieve vastly different release set points (*29*, *34*, *53*). The mammalian AZ is a highly resilient, redundant structure where the loss of a single component rarely abolishes core release functions entirely (*5*, *7*–*9*); consistent with this, the total abundance of Ca_V_2 channels (CAC) is only modestly reduced in *rim* mutants at both inputs. Because tonic MN-Ib synapses rely predominantly on modulating Ca^2+^ influx to tune release (*28*–*30*), they remain largely insensitive to the loss of *rim*. In contrast, phasic MN-Is synapses depend heavily on a large, tightly coupled readily releasable vesicle pool to sustain high *P*_r_, and our nanoscale imaging reveals that RIM is uniquely enriched at MN-Is, where it positions the priming factor Unc13A close to Ca_V_2 channel nanodomains. This reliance on RIM to maintain tight Ca_v_-vesicle coupling mirrors the mammalian Calyx of Held, a giant, high-fidelity synapse where channel-vesicle topography governs release precision (*51*) and RIM is essential for the tight coupling underlying rapid, synchronous release (*7*, *13*).

An important corollary is that MN-Ib’s robustness to loss of *rim* should not be read as evidence that scaffolding is dispensable at this input. AZ scaffolds are functionally redundant both within and across protein families, and several candidates could support release at MN-Ib in the absence of RIM. First, *Drosophila* encodes Fife, a Piccolo-RIM–related scaffold that promotes neurotransmitter release and organizes the AZ cytomatrix and vesicle clustering at this NMJ (*22*, *23*); although Fife shares RIM’s domain organization, loss of *fife*, but not *rim*, markedly disorganizes the AZ cytomatrix and vesicle clustering, indicating overlapping but nonidentical functions. Second, RIM acts in concert with RBP and ELKS/BRP to cluster Ca_V_2 channels and position priming factors (*9*, *10*), and both RBP and BRP have unusually prominent roles at the fly NMJ relative to many vertebrate synapses; consistent with a compensatory contribution, we find higher BRP and RBP levels at MN-Ib than MN-Is (Fig. 2). These scaffolds may carry disproportionate weight in the Ca^2+^ influx–dependent strategy of tonic terminals, offering a parsimonious explanation for why MN-Ib transmission is buffered against loss of *rim*. This logic parallels the mammalian literature, in which removing RIM1α alone produces mild phenotypes while additional loss of other isoforms reveals much stronger deficits (*9*, *54*), underscoring the fact that the apparent dispensability of a single scaffold gene often reflects redundancy rather than an absence of requirement for scaffolding per se.

The input-specific reliance on RIM extends into homeostatic plasticity. Foundational studies previously identified RIM as an essential effector of PHP, driving the homeostatic expansion of the RRP (*26*); by isolating inputs, we resolve a critical confound in those blended recordings, finding that RIM is required for acute PHP at phasic MN-Is, yet dispensable for chronic PHP at tonic MN-Ib. The selectivity aligns with the mechanisms of the two forms of plasticity—chronic PHP at MN-Ib is driven by enhanced Ca^2+^ influx, whereas acute PHP at MN-Is expands the RRP without altering Ca^2+^ influx (*28*). We further identify RIM as a coordinator of the homeostatic nanoscale reorganization of Ca_V_2 that accompanies acute PHP. Our STED measurements report the apparent area of epitope-defined CAC clusters rather than molecular density; the reduction in apparent area we observe is therefore consistent with, but does not establish, increased molecular packing, and lateral redistribution, altered molecular abundance, or differences in epitope accessibility could each contribute. Previous single-molecule studies have described analogous PHP-associated changes in active-zone organization as compaction (*36*, *49*, *55*). Although our STED measurements cannot determine whether the same process underlies the area changes reported here, one interpretation consistent with both datasets is that RIM promotes lateral reorganization and tighter release-site coupling of Ca_V_2 channels and associated priming machinery rather than a net increase in channel abundance. One attractive mechanism is liquid-liquid phase separation: RIM and RBP can condense into a dense phase that clusters Ca_V_2 channels and priming factors (*56*, *57*), which could allow RIM to homeostatically reorganize AZ nanostructure and reposition Unc13A nearer Ca_V_2, recruiting additional loosely coupled vesicles into the releasable pool, consistent with our EGTA-sensitivity assays. Whether this RIM-dependent reorganization is stable or reversible remains open: Because acute PHP is induced here with the poorly reversible antagonist PhTx, reversibility cannot be assessed on the timescale of our measurements, but future work using a reversible glutamate-receptor antagonist (e.g., GYKI) could test whether the reorganization relaxes upon restoration of receptor function (*58*).

The specialization of RIM at the fly NMJ offers a framework for interpreting emerging evidence of AZ heterogeneity in the mammalian brain. Whereas *Drosophila* uses a single *rim* gene to differentially tune convergent inputs, the mammalian genome encodes multiple *rim* genes and isoforms (RIM1α, RIM1β, and RIM2) with synapse-specific expression and functions (*54*, *59*). This expansion may have evolved in part to fulfill analogous tunable, input-specific roles, allowing circuits to independently set *P*_r_, short-term plasticity, and long-term plasticity across vast numbers of heterogeneous connections. Consistent with this, RIM’s requirements vary markedly by synapse type: it supports docking, priming, and Ca^2+^ channel tethering at classical fast synapses (*7*, *16*), yet operates within a sparse, specialized machinery at dopaminergic terminals (*17*, *18*, *21*), and is required for presynaptic long-term potentiation at hippocampal mossy fibers (*60*). Such heterogeneity underscores a potentially conserved principle—synapses built for high-demand signaling or rapid plasticity rely more heavily on dedicated protein modules—whereby deploying RIM in proportion to the demand for Ca_v_-vesicle coupling lets diverse circuits set their baseline *P*_r_ and dynamic range, perhaps by exploiting the biophysical tunability of LLPS condensates to adjust the local organization of release machinery across distinct synapse subtypes (*57*, *61*, *62*).

Last, our finding that a core AZ scaffold like RIM acts as a tunable, input-specific module invites a re-evaluation of active-zone architecture. The AZ is built from a conserved protein network (ELKS/BRP, RBP, Liprin-α, Piccolo/Bassoon/Fife, Syd-1, and Unc13) (*5*, *6*, *8*, *10*), yet much of the foundational work characterizing these components at the *Drosophila* NMJ relies on blended recordings that may mask input-specific specializations like those described here. Just as RIM is specialized for the high-*P*_r_ phasic input, other components—specific Unc13 isoforms, RBP, or BRP—may prove disproportionately important for the Ca^2+^ influx–dependent strategy of tonic MN-Ib (*39*, *50*). Together with the growing literature on active-zone molecular diversity (*1*, *3*, *4*), these findings support a view of AZs not as invariant relay points but as molecularly diverse structures whose composition shapes the input-specific transformation of activity into transmitter release.

## MATERIALS AND METHODS

### Fly stocks

*Drosophila* stocks were raised at 25°C on standard molasses food. Unless otherwise specified, *w^1118^* served as the wild-type control and genetic background for all genotypes. For input-specific silencing, we crossed *UAS-BoNT-C* with *Is-GAL4* (*GMR27E09-GAL4*) or *Ib-GAL4* (*dHb9-GAL4*) (*35*). We used the endogenously tagged *cac^sfGFP-N^* to visualize CAC (*47*) and *GluRIIA^PV3^* mutants to induce chronic PHP (*28*). All experiments used third-instar larvae of both sexes. See table S2 for a complete list of fly stocks, reagents, and software.

### Molecular biology

The *rim^ALFA^* allele was generated by WellGenetics (Taipei, Taiwan) via CRISPR-Cas9–mediated homology-directed repair (HDR). A guide RNA (gRNA) targeting the beginning of the *rim* coding sequence near the start codon was cloned into a U6 promoter plasmid. A donor plasmid (pUC57-Kan backbone) was constructed to encode an ALFA tag (*37*) inserted immediately after the ATG start codon, a 3xP3-DsRed selection cassette flanked by piggybac terminal repeats, and ∼1 kb upstream and downstream homology arms amplified from the injection strain. Silent PAM mutations were introduced into the donor to prevent Cas9 re-cleavage following HDR. The gRNA, *hs-Cas9*, and donor plasmids were co-injected into *w^1118^* embryos. F1 progeny were screened for 3xP3-DsRed fluorescence. Correct insertion at the *rim* locus was verified by using junction polymerase chain reaction (PCR) and Sanger sequencing. Donor backbone integration was excluded by integration-specific PCR. The PiggyBac-flanked selection cassette was subsequently excised, leaving a TTAA footprint between the ALFA tag and the *rim* coding sequence; excision was confirmed by PCR and sequencing.

### Electrophysiology

All dissections and sharp-electrode current clamp recordings were performed as previously described (*28*). Preparations were bathed in a modified hemolymph-like saline (HL-3) containing (in millimolar): 70 NaCl, 5 KCl, 10 MgCl_2_, 10 NaHCO_3_, 115 sucrose, 5 trehalose, 5 Hepes, and 0.4 CaCl_2_ (pH 7.2). Recordings were acquired on an Olympus BX61 WI microscope equipped with a 40×/0.80 NA water-dipping objective, using an Axoclamp 900A amplifier, Digidata 1440A acquisition system, and pClamp 10.5 software (Molecular Devices). Data were analyzed only from cells with initial resting potentials more hyperpolarized than −60 mV and input resistances of >5 MΩ. All recordings were performed on abdominal muscle 6 or 7 in segments A2, A3, or A4 of third-instar larvae. mEPSPs were recorded for 60 s in the absence of any stimulation and analyzed using MiniAnalysis (Synaptosoft) and Excel (Microsoft) software. The average mEPSP amplitude for each NMJ was calculated from ∼100 consecutive events. EPSPs were evoked by delivering 20 stimuli (0.5 ms duration, 0.5 Hz) to the motor nerve via an ISO-Flex stimulus isolator (A.M.P.I.) at intensities required to recruit both motor axons. Two-electrode voltage-clamp recordings were performed as previously described (*28*, *29*). Larvae were bathed in HL-3 containing the indicated CaCl_2_ concentrations. Recordings were acquired on the same microscope and amplifier described above. We recorded from abdominal muscle 6 (segments A3 and A4), requiring an initial resting potential below −60 mV and an input resistance >5 MΩ. EPSCs were evoked by delivering 20 stimuli (0.5 ms duration, 0.5 Hz) via an ISO-Flex stimulus isolator. To acutely block postsynaptic receptors and induce PHP, semidissected larvae (dorsal incision only) were incubated in HL-3 containing 20 μM PhTx-433 (Sigma-Aldrich) for 12 min (*63*). Preparations were then washed three times with HL-3 prior to recording.

To estimate the RRP size, we evoked EPSCs with a 30-stimulus, 60-Hz train and calculated the cumulative EPSC (the running sum of evoked EPSC amplitudes across the train). A linear fit to stimuli 19 to 30 was back-extrapolated to time 0, and this *y* intercept was divided by the mean mEPSP amplitude to estimate RRP size. The slope of this fit was taken as the steady-state vesicle recruitment rate. RRP estimates were obtained primarily in 3 mM extracellular Ca^2+^, which drives the near-complete synaptic depression required for reliable back-extrapolation; this approach is not applicable to the facilitating MN-Ib input. To confirm the key findings under physiological conditions, we repeated these measurements at MN-Is in 1.5 mM Ca^2+^ at baseline and after PhTx (table S1). To evaluate EGTA sensitivity, we incubated larval fillets in 0 Ca^2+^ HL-3 supplemented with 50 μM EGTA-AM (Sigma-Aldrich) for 10 min, followed by three washes with standard HL-3 prior to recording (*28*). Where indicated, EGTA-AM was applied following 10-min PhTx incubation.

### Immunocytochemistry

Third-instar larvae were dissected in ice-cold 0 Ca^2+^ HL-3 and immunostained as previously described (*64*). Preparations were fixed in ice-cold 100% methanol (5 min), Bouin’s fixative (5 min), or 4% PFA (10 min), followed by three 10-min washes in PBST (PBS + 0.1% Triton X-100). Samples were blocked in 5% Normal Donkey Serum, incubated with primary antibodies overnight at 4°C, washed 3× in PBST, and incubated with secondary antibodies for 2 hours at room temperature. Following three final PBST washes, samples were equilibrated in 70% glycerol and mounted in VectaShield for confocal imaging or ProLong Glass (Thermo Fisher Scientific) for STED microscopy. CAC (*cac^sfGFP-N^*) was detected using a mouse anti-GFP primary antibody rather than by native sfGFP fluorescence in STED experiments. Primary antibodies included mouse anti-BRP (nc82, 1:250), mouse anti-GFP (1:400), guinea pig anti-RBP (1:1000), rabbit anti-Unc13A (1:1000), guinea pig anti-Unc13B (1:500), rabbit anti-ALFA (1:500), and Alexa Fluor 647–conjugated goat anti-HRP (1:400). Secondary antibodies included STAR RED–conjugated secondaries for STED imaging (1:200) and Cy3-, AF488-, AF594-, or AF647-conjugated secondaries (1:400). See table S2 for complete details and sources. To ensure robust PhTx-induced remodeling for imaging experiments, we used a modified protocol using 80 μM PhTx for 15 min. Preparations were co-stained with BRP to verify consistent remodeling.

### Confocal imaging and analysis

Confocal images were acquired on a Nikon A1R microscope (100× APO 1.40 NA oil objective) using NIS Elements 4.51 software and 405, 488, 561, and 647 nm laser lines (*65*). For intensity quantification of BRP, RBP, CAC, Unc13A, Unc13B, and RIM, *z*-stacks were collected at 0.125 μm *x*-*y* pixel size and 0.15 μm *z*-step with single-pass (1×) line scanning. Antigens were imaged in co-stained sets, each including BRP as a common active-zone reference and anti-HRP to label the neuronal membrane: (i) BRP, RBP, and RIM (detected via the ALFA tag); (ii) CAC (*cac^sfGFP-N^*) and BRP; and (iii) Unc13A, Unc13B, and BRP. Within each experiment, laser power and detector gain were set on wild-type control samples at the start of the imaging session to span the dynamic range without pixel saturation and were held constant across all genotypes imaged in that session. For antigens stained in sessions subsequent to the original experiment, wild-type controls were re-stained and re-imaged in the same session, and mutant measurements were normalized to the within-session wild-type mean.

Image analysis was performed in NIS Elements 4.51 on maximum-intensity projections of individual boutons. MN-Ib and MN-Is boutons were distinguished by anti-HRP morphology and bouton size, and a region of interest for each bouton was defined from the anti-HRP signal. Within each region, active-zone puncta were identified using a fixed intensity threshold determined on wild-type boutons at the start of each imaging session and applied uniformly to all images acquired in that session; only puncta colocalizing with the BRP and/or CAC active-zone reference signal, judged by visual overlap of the merged channels, were quantified. Analysis was restricted to boutons located near the center of the field of view, where illumination is uniform; boutons that were saturated, incompletely captured within the *z*-stack, torn, or showed obvious staining artifact were excluded. The mean fluorescence intensity of the included puncta was averaged to yield a single value per bouton, and no background subtraction was applied. For input comparisons (Fig. 2), per-NMJ MN-Is intensity was normalized to the pooled within-session wild-type MN-Ib mean and expressed as a percentage of MN-Ib; for genotype comparisons (Fig. 3), mutant values were normalized to the pooled within-session wild-type mean for the corresponding input. Absolute fluorescence values are reported in table S1. Measurements were obtained from muscle 6/7 terminal boutons (segments A3 to A5). Each data point represents a single MN-Ib bouton or a small group of one to three MN-Is boutons averaged together, with the mean intensity of all active-zone puncta within averaged to yield that value; the bouton (or MN-Is bouton group) served as the statistical unit. Data were obtained from more than 10 NMJs and more than four animals per genotype.

### STED imaging and quantification

STED microscopy was performed on an Abberior STEDYCON system mounted on a Nikon Eclipse FN1 microscope (*66*), equipped with 640, 561, 488, and 405 nm excitation lasers, a 775 nm pulsed depletion laser, and three avalanche photodiode detectors. Two-color images (one to two boutons per field) were acquired with a 100× Plan APO 1.45 NA oil-immersion objective at 20 nm pixel size, 10 μs dwell time, and 15× line accumulation. Abberior STAR RED and Alexa Fluor 594 were depleted at 775 nm, time-gated at 1 ns (6 ns width), and detected sequentially (STAR RED, 675 nm; AF594, 600 nm). Because native sfGFP is not depleted at 775 nm, CAC (*cac^sfGFP-N^*) was detected with a mouse anti-GFP primary and a STAR RED–conjugated secondary antibody. For distance measurements, the detected epitopes were CAC, the N-terminal sfGFP knock-in (*cac^sfGFP-N^*); RIM, the N-terminal ALFA tag near the zinc-finger domain; Unc13A and Unc13B, the indicated C-terminal antibodies; and BRP, the nc82 epitope; absolute interprotein distances should be interpreted in light of these positions.

All images were deconvolved in SVI Huygens (classic maximum-likelihood estimation, theoretical STED point-spread function, per-channel signal-to-noise estimation, 27 iterations), and all quantification was performed on the deconvolved images. To minimize projection artifacts from tilted AZs, analysis was restricted to planar, en-face AZs near the center of boutons, excluding those at the lateral edges. Planarity was judged from the co-imaged reference channel: a closed, approximately circular BRP T-bar ring where BRP was imaged, or a compact, circular en-face CAC cluster in two-color acquisitions lacking BRP. Identical selection criteria were applied across all genotypes and conditions.

Interprotein distances were measured on deconvolved 16-bit images by drawing a 1-pixel-wide line through the intensity maxima of the two puncta (ImageJ line-profile tool) and taking the peak-to-peak separation, converted to nanometers (one measurement per AZ). CAC cluster area was quantified in NIS Elements (v4.51, General Analysis) from a per-punctum binary mask generated by manual thresholding of signal-bearing pixels (preprocessing: Smooth 1×, Clean 3×, Fill Holes); this analysis was performed entirely on genotype- and condition-coded files by an analyst blinded with a randomized key. RIM puncta per BRP ring were counted with the ImageJ Find Maxima function (prominence = 1200 gray levels, empirically set to detect RIM puncta above background and held constant across genotypes/conditions) on deconvolved images. For representative panels, each deconvolved image is presented in two display versions: an unsmoothed rendering and a display-only version generated by bicubic interpolation (Adobe Photoshop) for visual clarity. The apparent pixel dimensions in the displayed figure panels should not be interpreted as the 20-nm pixel sampling used during image acquisition. Only uniform linear brightness/contrast adjustments were applied equivalently across all compared conditions; no nonlinear intensity adjustments were made, and the interpolated images were used solely for display, never for quantification. All measurements were obtained from two-dimensional images of muscle 6/7 NMJs (segments A3 to A5); each data point represents one AZ, from ≥4 animals per condition.

### Statistical analysis

Statistical analyses were performed in Python using SciPy and statsmodels; graphs were prepared in GraphPad Prism (v8.0), and event/image quantification used MiniAnalysis, NIS Elements, SVI Huygens Essential (v22.10), and Excel. For electrophysiology, *n* represents individual NMJs; for confocal and STED imaging, *n* represents individual boutons or AZs, respectively. Normality was assessed by the Shapiro-Wilk test and homogeneity of variance by the Brown-Forsythe test. Normally distributed data were compared by unpaired two-tailed Welch’s *t* test. Ratiometric measures (quantal content, paired-pulse ratio, EPSC amplitude, and mean fluorescence intensity) were log-transformed, and failure rate was arcsine-square-root transformed, prior to testing; data that could not be normalized by transformation were compared by Mann-Whitney *U* test and are reported as median with interquartile range. Fluorescence-intensity comparisons (Figs. 2 and 3) were each treated as an independent, prespecified single-protein comparison and were not corrected for multiple comparisons. Families of planned two-group comparisons—wild-type versus *rim* across inputs (Fig. 1), across extracellular [Ca^2+^] and for paired-pulse ratio and failure rate (fig. S1), EGTA sensitivity (Fig. 7, A and B), and CAC-Unc13A distance ± PhTx (Fig. 7, C and D)—were corrected by the Holm-Šidák method, applied within but not across distinct measures; wild-type versus the two *rim*-null alleles (fig. S2, C to F) used single-step Šidák. Factorial designs were analyzed by two-way analysis of variance (ANOVA) with genotype and a second factor (extracellular [Ca^2+^], PhTx, *GluRIIA* background, or EGTA); the genotype × factor interaction tested whether an effect (homeostatic potentiation, calcium dependence, or EGTA sensitivity) differed between genotypes. For the homeostatic-potentiation experiments (Fig. 4 and fig. S2, H to K), the CAC cluster-area analysis (Fig. 6, G and H), and the cumulative EPSC, estimated RRP size, and recruitment-rate analyses (Fig. 5), within-genotype comparisons of the treated condition to baseline were made by unpaired two-tailed Welch’s *t* test (log-transformed where required) with single-step Šidák correction; the corresponding between-genotype baseline comparisons in Fig. 5 used Welch’s *t* test or Mann-Whitney *U* as above. The calcium-series (fig. S1) and EGTA (Fig. 7, A and B) analyses used Holm-Šidák–corrected pairwise comparisons. Non-normally distributed factorial data (CAC-Unc13A distances; Fig. 7, C and D) were evaluated for interaction by aligned rank transform (*67*), implemented in Python (statsmodels), with Mann-Whitney *U* post hoc comparisons corrected by Holm-Šidák (*67*). Error bars indicate SEM; significance is denoted by *P < 0.05, **P < 0.01, ***P < 0.001, ****P < 0.0001, and ns (not significant). Complete statistical details for all datasets are provided in table S1.

### Rigor and reproducibility

Acquisition and analysis used standardized, predefined settings to limit experimenter bias, as described above for confocal, STED, and electrophysiological experiments. Primary data acquisition and quantification were not performed blind to genotype or condition, with two exceptions. First, CAC cluster area at MN-Is (Fig. 6, G and H) was quantified entirely from genotype- and condition-coded files by an analyst masked to condition. Second, an additional Unc13A/Unc13B intensity dataset at MN-Ib and MN-Is (wild type and *rim* null) was acquired and analyzed under genotype-blinding; this independent dataset reproduced the significant reduction in Unc13A at MN-Is in *rim* mutants reported in Fig. 3. Remaining datasets were acquired and analyzed without formal blinding.

## Supplementary Materials

### This PDF file includes:

Figs. S1 and S2 Tables S1 and S2 References
