# Supplementary material for: Input-specific deployment of the active zone scaffold RIM tunes synaptic release probability and plasticity
Source: Sci Adv. 2026 Nov 11;12(46):eaeh4857. doi: 10.1126/sciadv.aeh4857 (PMC13754025; doi:10.1126/sciadv.aeh4857)
Supplement: Supplementary file 1 [file sciadv.aeh4857_sm.pdf]

Science Advances
AAAS

Supplementary Materials for

# Input-specific deployment of the active zone scaffold RIM tunes synaptic release probability and plasticity

Rebecca Stark *et al.*

Corresponding author: Dion Dickman, dickman@usc.edu

*Sci. Adv.* **12**, eaeh4857 (2026)
DOI: 10.1126/sciadv.aeh4857

**This PDF file includes:**

Figs. S1 and S2
Tables S1 and S2
References

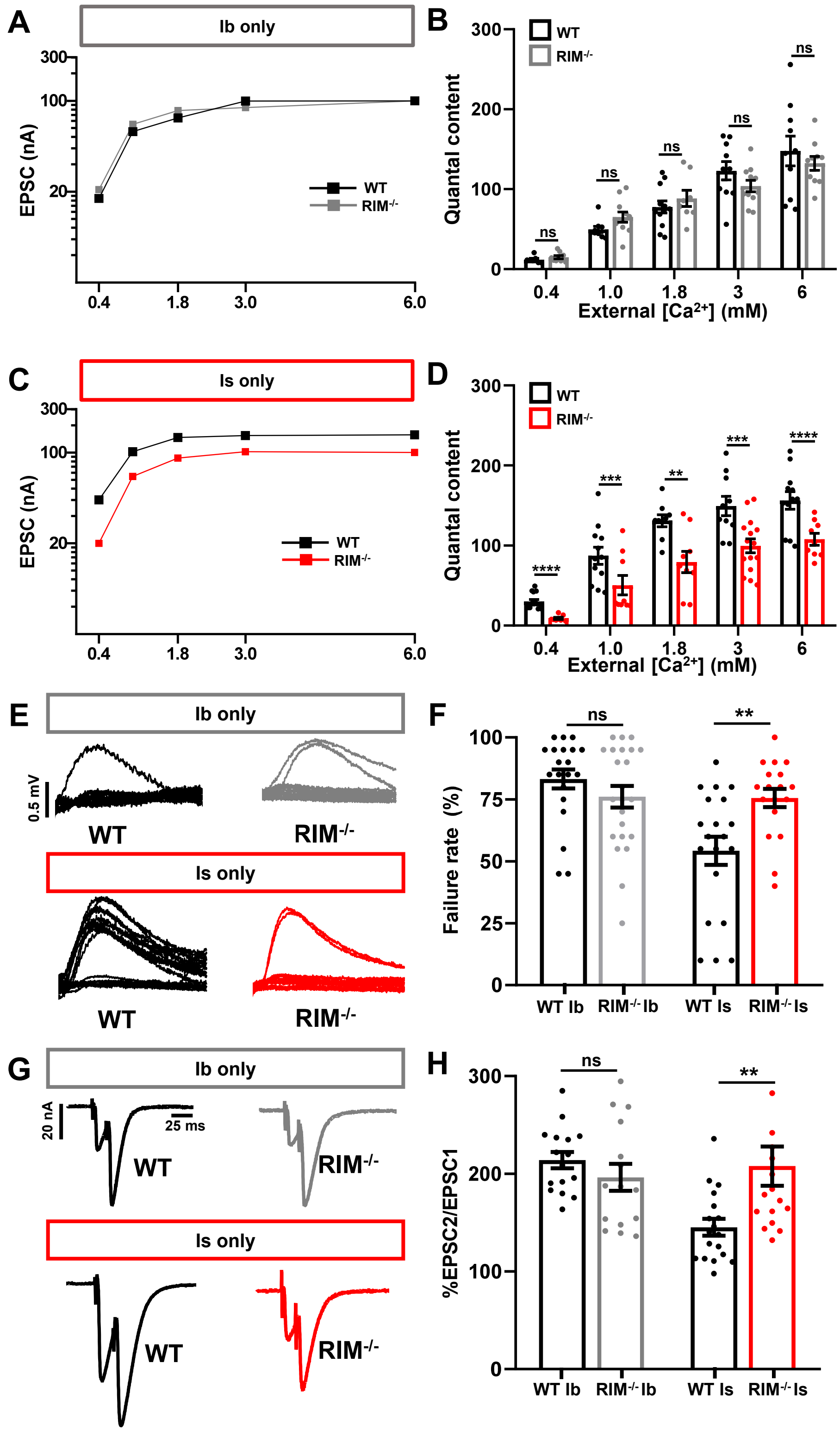


**Figure S1: Additional electrophysiological analyses confirm loss of *rim* only impacts transmission at MN-Is. (A,B)** Plot of EPSC values as a function of external [$Ca^{2+}$] (A) and quantification of quantal content (B) for isolated MN-Ib in wild-type and *rim*$^{-/-}$ synapses. No differences are observed in *rim* mutants. **(C,D)** Corresponding data for isolated MN-Is, revealing clear reductions in *rim* mutants. **(E,F)** Representative traces (E) and quantification of failure rate (F) across 20 stimuli (0.5 Hz) at 0.10 mM extracellular [$Ca^{2+}$], confirming lowered release probability selectively at *rim*$^{-/-}$ MN-Is. **(G,H)** Representative paired-pulse EPSC traces (G) and quantification of paired-pulse ratios (EPSC2/EPSC1) (H) at 1.8 mM extracellular [$Ca^{2+}$] (16.7 msec interstimulus interval), revealing deficits in release probability only at *rim*$^{-/-}$ MN-Is. Error bars indicate ±SEM. Additional statistical details are provided in Table S1.

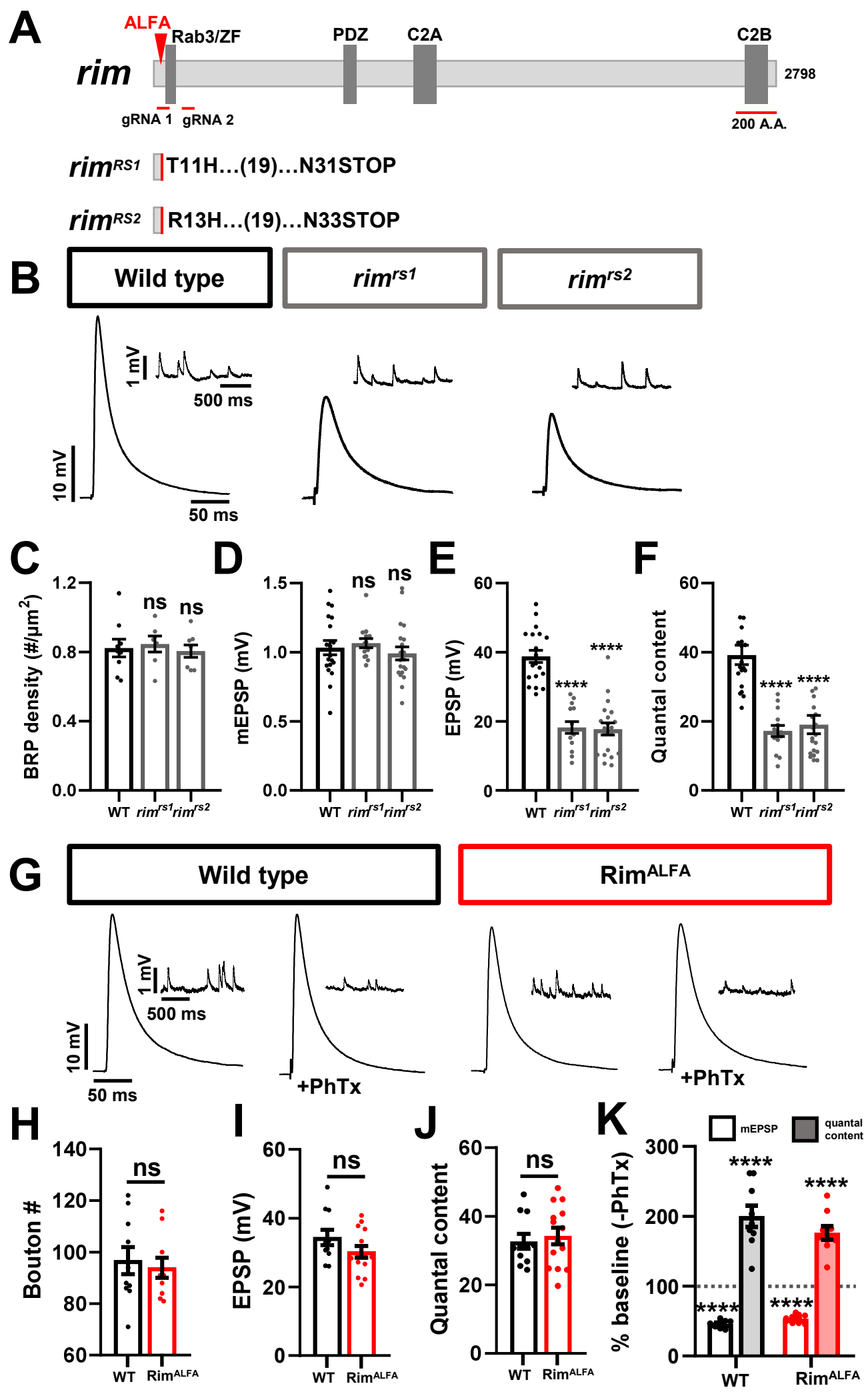


**Figure S2: Generation and validation of *rim* null alleles and the *rim*$^{ALFA}$ tag. (A)** Schematic of the Drosophila *rim* locus indicating sgRNA targets for CRISPR mutagenesis and the ALFA tag insertion site, along with the resulting mutant alleles (*rim*$^{RS1}$ *and rim*$^{RS2}$) and early stop codons. **(B)** Representative mEPSP and EPSP traces from wild type, *rim*$^{RS1}$, and *rim*$^{RS2}$ at baseline. **(C)** Quantification of BRP density at MN-Ib boutons, revealing no change in active zone number in *rim* mutants. **(D)** Quantification of mEPSP amplitude, indicating normal spontaneous release in *rim*$^{RS1}$ and *rim*$^{RS2}$ mutants. **(E,F)** Quantification of evoked EPSP amplitude and quantal content, revealing a ~50% reduction in evoked release in both *rim* null alleles, consistent with previously reported *rim* null alleles (15, 26). Wild type and *rim*$^{RS1}$ data in panels B–F are also presented in Figure 1. **(G)** Representative mEPSP and EPSP traces from wild type and *rim*$^{ALFA}$ animals at baseline and following PhTx application. **(H-K)** Quantification of bouton number, EPSP amplitude, quantal content, and PHP expression, revealing no changes in synaptic growth, transmission, or presynaptic homeostatic potentiation in *rim*$^{ALFA}$ animals. Error bars indicate ±SEM. Additional statistical details are provided in Table S1.

**Table S1: Absolute values for normalized data and additional statistical details.** The figure and panel, genotype, and other conditions are noted. Average values (with standard error of the mean noted in parentheses), data samples (n), and statistical significance tests are shown for all data.

| Figure | Label | [$Ca^{2+}$] (mM) | Genotype | PhTx | mEPSP amplitude (mV) | EPSP amplitude (mV)/EPSC (nA) | QC | mEPSP frequency (Hz) | $R_{input}$ (MΩ) | Resting potential (mV) | n | WT vs. *rim* at each condition by unpaired two-tailed Welch's t-test, Holm-Šidák corrected. P value (significance): mEPSP, EPSP/EPSC, QC. |
|---|---|---|---|---|---|---|---|---|---|---|---|---|
| 1D - E | wild type (Ib+Is) | 0.4 | *$w^{1118}$* | - | 1.03 (±0.05) | 38.80 (±1.79) | 39.21 (±2.81) | 4.22 (±0.52) | 7.42 (±0.51) | -70.25 (±1.51) | 19 | - |
| 1D - E | RIM$^{-/-}$ (Ib+Is) | 0.4 | *w;+;$rim^{RS1}$* | - | 1.07 (±0.03) | 18.23 (±1.72) | 17.22 (±1.62) | 2.66 (±0.64) | 6.43 (±0.63) | -68.00 (±1.57) | 14 | 0.9379 (ns), <0.0001 (****), <0.0001 (****) |
| 1D - E | WT Ib only | 0.4 | *w;+;Is-Gal4/UAS-BoNT-C* | - | 0.81 (±0.05) | 9.68 (±1.25) | 12.68 (±2.08) | 3.16 (±0.36) | 7.77 (±0.64) | -66.09 (±1.42) | 15 | - |
| 1D - E | RIM$^{-/-}$ Ib only | 0.4 | *w;+;$rim^{RS1}$,Is-GAL4/ $rim^{RS1}$,UAS-BoNT-C* | - | 0.81 (±0.05) | 9.97 (±1.08) | 12.92 (±1.53) | 3.42 (±0.32) | 7.20 (±0.60) | -66.58 (±1.53) | 14 | 0.9945 (ns), 0.8599 (ns), 0.7803 (ns) |
| 1D - E | WT Is only | 0.4 | *w;+;Ib-GAL4/UAS-BoNT-C* | - | 1.19 (±0.06) | 26.09 (±1.38) | 22.70 (±1.78) | 2.89 (±0.23) | 7.33 (±0.59) | -64.97 (±0.79) | 15 | - |
| 1D - E | RIM$^{-/-}$ Is only | 0.4 | *w;+;$rim^{RS1}$,Ib-GAL4/$rim^{RS1}$,UAS-BoNT-C* | - | 1.16 (±0.04) | 10.25 (±1.27) | 8.73 (±0.90) | 3.74 (±0.50) | 7.78 (±0.78) | -65.26 (±1.02) | 9 | 0.9379 (ns), <0.0001 (****), <0.0001 (****) |
| 1D - E | wild type (Ib+Is) | 1.8 | *$w^{1118}$* | - | 1.04 (±0.07) | 184.97 (±10.55) | 185.87 (±13.07) | 2.90 (±0.41) | 7.07 (±0.81) | -- | 15 | - |
| 1D - E | RIM$^{-/-}$ (Ib+Is) | 1.8 | *w;+;$rim^{RS1}$* | - | 1.00 (±0.07) | 139.56 (±4.67) | 144.98 (±6.76) | 4.04 (±0.58) | 8.64 (±0.77) | -- | 14 | 0.8910 (ns), 0.0017 (**), 0.0224 (*) |
| 1D - E | WT Ib only | 1.8 | w;+;Is-GAL4/*UAS-BoNT-C* | - | 0.87 (±0.07) | 64.67 (±5.06) | 77.55 (±7.50) | 3.19 (±0.36) | 6.25 (±0.58) | -- | 12 | - |
| 1D - E | RIM$^{-/-}$ Ib only | 1.8 | *w;+;$rim^{RS1}$,Is-GAL4/$rim^{RS1}$,UAS-BoNT-C* | - | 0.91 (±0.08) | 76.41 (±6.64) | 88.46 (±10.17) | 3.02 (±0.69) | 7.33 (±0.75) | -- | 8 | 0.8910 (ns), 0.1809 (ns), 0.4028 (ns) |
| 1D - E | WT Is only | 1.8 | *w;+;Ib-GAL4/UAS-BoNT-C* | - | 1.12 (±0.04) | 146.97 (±8.89) | 131.14 (±7.47) | 4.43 (±0.62) | 7.11 (±0.95) | -- | 9 | - |
| 1D - E | RIM$^{-/-}$ Is only | 1.8 | *w;+;$rim^{RS1}$,Ib-GAL4/$rim^{RS1}$,UAS-BoNT-C* | - | 1.19 (±0.10) | 86.89 (±9.89) | 79.35 (±13.21) | 1.62 (±0.26) | 7.22 (±0.78) | -- | 9 | 0.8910 (ns), 0.0011 (**), 0.0143 (*) |

| Figure | Label | [$Ca^{2+}$] (mM) | Genotype | mEPSP amplitude (mV) | EPSC (nA) | QC | mEPSP frequency (Hz) | $R_{input}$ (MΩ) | n | WT vs. rim at each [$Ca^{2+}$] by two-way ANOVA (genotype × [$Ca^{2+}$]) with post-hoc Welch's t-test, Holm-Šidák corrected. P value (significance): mEPSP, EPSC, QC. |
|---|---|---|---|---|---|---|---|---|---|---|
| *Additional data* | wild type (Ib+Is) | 0.4 | *$w^{1118}$* | 1.08 (±0.05) | 46.48 (±3.28) | 43.62 (±3.21) | 4.36 (±0.36) | 7.50 (±0.61) | 16 | - |
| *Additional data* | RIM$^{-/-}$ (Ib+Is) | 0.4 | *w;+;$rim^{RS1}$* | 1.07 (±0.04) | 18.12 (±1.99) | 16.84 (±1.67) | 4.60 (±0.48) | 7.85 (±0.69) | 13 | 0.9972 (ns), <0.0001 (****), <0.0001 (****) |
| S1A - D | WT Ib only | 0.4 | *w;+;Is-Gal4/UAS-BoNT-C* | 0.74 (±0.05) | 8.44 (±0.78) | 11.88 (±1.64) | 3.41 (±0.48) | 9.14 (±0.88) | 7 | - |
| S1A - D | RIM$^{-/-}$ Ib only | 0.4 | *w;+;$rim^{RS1}$,Is-GAL4/ $rim^{RS1}$,UAS-BoNT-C* | 0.73 (±0.04) | 10.54 (±1.01) | 14.96 (±1.92) | 2.86 (±0.47) | 8.33 (±0.83) | 9 | 0.9986 (ns), 0.3755 (ns), 0.6460 (ns) |
| S1A - D | WT Is only | 0.4 | *w;+;Ib-GAL4/UAS-BoNT-C* | 1.26 (±0.05) | 30.13 (±2.44) | 24.29 (±1.96) | 4.12 (±0.27) | 7.87 (±0.77) | 15 | - |
| S1A - D | RIM$^{-/-}$ Is only | 0.4 | *w;+;$rim^{RS1}$,Ib-GAL4/$rim^{RS1}$,UAS-BoNT-C* | 1.12 (±0.04) | 9.99 (±1.37) | 9.00 (±1.29) | 3.86 (±0.50) | 7.62 (±0.82) | 8 | 0.1376 (ns), <0.0001 (****), <0.0001 (****) |

| | | | | | | | | | | |
|---|---|---|---|---|---|---|---|---|---|---|
| *Additional data* | wild type (Ib+Is) | 1 | *$w^{1118}$* | 1.18 (±0.08) | 128.10 (±7.27) | 118.42 (±13.26) | 2.64 (±0.20) | 7.86 (±0.77) | 14 | - |
| *Additional data* | RIM$^{-/-}$ (Ib+Is) | 1 | *w;+;$rim^{RS1}$* | 1.16 (±0.03) | 94.10 (±6.39) | 82.33 (±6.68) | 3.01 (±0.21) | 7.08 (±0.89) | 12 | 0.9972 (ns), 0.0023 (**), 0.0532 (ns) |
| S1A - D | WT Ib only | 1 | *w;+;Is-Gal4/UAS-BoNT-C* | 0.94 (±0.05) | 45.72 (±2.90) | 49.75 (±3.86) | 2.63 (±0.27) | 5.78 (±0.36) | 9 | - |
| S1A - D | RIM$^{-/-}$ Ib only | 1 | *w;+;$rim^{RS1}$,Is-GAL4/ $rim^{RS1}$,UAS-BoNT-C* | 0.87 (±0.05) | 54.87 (±4.44) | 65.72 (±7.03) | 3.14 (±0.28) | 7.00 (±0.70) | 10 | 0.9593 (ns), 0.3755 (ns), 0.3448 (ns) |
| S1A - D | WT Is only | 1 | *w;+;Ib-GAL4/UAS-BoNT-C* | 1.22 (±0.07) | 102.07 (±10.27) | 87.29 (±10.57) | 2.63 (±0.44) | 5.25 (±0.18) | 12 | - |
| S1A - D | RIM$^{-/-}$ Is only | 1 | *w;+;$rim^{RS1}$,Ib-GAL4/$rim^{RS1}$,UAS-BoNT-C* | 1.32 (±0.14) | 54.58 (±6.73) | 50.45 (±12.17) | 2.11 (±0.29) | 5.56 (±0.56) | 9 | 0.8910 (ns), 0.0019 (**), 0.0280 (*) |
| *Additional data* | wild type (Ib+Is) | 1.8 | *$w^{1118}$* | 1.04 (±0.07) | 184.97 (±10.55) | 185.87 (±13.07) | 2.90 (±0.41) | 7.07 (±0.81) | 15 | - |
| *Additional data* | RIM$^{-/-}$ (Ib+Is) | 1.8 | *w;+;$rim^{RS1}$* | 1.00 (±0.07) | 139.56 (±4.67) | 144.98 (±6.76) | 4.04 (±0.58) | 8.64 (±0.77) | 14 | 0.9780 (ns), 0.0019 (**), 0.0532 (ns) |
| S1A - D | WT Ib only | 1.8 | *w;+;Is-Gal4/UAS-BoNT-C* | 0.87 (±0.07) | 64.67 (±5.06) | 77.55 (±7.50) | 3.19 (±0.36) | 6.25 (±0.58) | 12 | - |
| S1A - D | RIM$^{-/-}$ Ib only | 1.8 | *w;+;$rim^{RS1}$,Is-GAL4/ $rim^{RS1}$,UAS-BoNT-C* | 0.91 (±0.08) | 76.41 (±6.64) | 88.46 (±10.17) | 3.02 (±0.69) | 7.33 (±0.75) | 8 | 0.9963 (ns), 0.3755 (ns), 0.6460 (ns) |
| S1A - D | WT Is only | 1.8 | *w;+;Ib-GAL4/UAS-BoNT-C* | 1.12 (±0.04) | 146.97 (±8.89) | 131.14 (±7.47) | 4.43 (±0.62) | 7.11 (±0.95) | 9 | - |
| S1A - D | RIM$^{-/-}$ Is only | 1.8 | *w;+;$rim^{RS1}$,Ib-GAL4/$rim^{RS1}$,UAS-BoNT-C* | 1.19 (±0.10) | 86.89 (±9.89) | 79.35 (±13.21) | 1.62 (±0.26) | 7.22 (±0.78) | 9 | 0.8910 (ns), 0.0052 (**), 0.0280 (*) |
| *Additional data* | wild type (Ib+Is) | 3 | *$w^{1118}$* | 1.02 (±0.06) | 213.23 (±9.19) | 221.99 (±15.45) | 3.69 (±0.47) | 9.75 (±0.52) | 20 | - |
| *Additional data* | RIM$^{-/-}$ (Ib+Is) | 3 | *w;+;$rim^{RS1}$* | 0.98 (±0.06) | 146.08 (±9.10) | 151.72 (±11.07) | 3.79 (±0.17) | 8.00 (±0.65) | 12 | 0.9835 (ns), 0.0003 (***), 0.0034 (**) |
| S1A - D | WT Ib only | 3 | *w;+;Is-Gal4/UAS-BoNT-C* | 0.83 (±0.03) | 99.38 (±6.95) | 122.92 (±11.38) | 3.13 (±0.46) | 7.70 (±0.67) | 10 | - |
| S1A - D | RIM$^{-/-}$ Ib only | 3 | *w;+;$rim^{RS1}$,Is-GAL4/ $rim^{RS1}$,UAS-BoNT-C* | 0.84 (±0.06) | 84.05 (±4.37) | 103.85 (±7.16) | 2.60 (±0.33) | 9.18 (±0.81) | 11 | 0.9986 (ns), 0.3755 (ns), 0.6460 (ns) |
| S1A - D | WT Is only | 3 | *w;+;Ib-GAL4/UAS-BoNT-C* | 1.06 (±0.06) | 153.74 (±8.24) | 149.32 (±12.06) | 3.61 (±0.56) | 9.20 (±0.44) | 10 | - |
| S1A - D | RIM$^{-/-}$ Is only | 3 | *w;+;$rim^{RS1}$,Ib-GAL4/$rim^{RS1}$,UAS-BoNT-C* | 1.05 (±0.06) | 102.09 (±8.48) | 99.65 (±8.70) | 1.70 (±0.24) | 9.40 (±0.68) | 15 | 0.9139 (ns), 0.0010 (***), 0.0071 (**) |
| *Additional data* | wild type (Ib+Is) | 6 | *$w^{1118}$* | 0.96 (±0.07) | 210.95 (±8.55) | 233.21 (±16.40) | 2.48 (±0.26) | 6.38 (±0.53) | 15 | - |
| *Additional data* | RIM$^{-/-}$ (Ib+Is) | 6 | *w;+;$rim^{RS1}$* | 1.07 (±0.06) | 145.59 (±10.49) | 139.61 (±12.30) | 2.40 (±0.27) | 7.88 (±1.20) | 14 | 0.6405 (ns), 0.0011 (**), 0.0016 (**) |
| S1A - D | WT Ib only | 6 | *w;+;Is-Gal4/UAS-BoNT-C* | 0.75 (±0.08) | 99.61 (±6.80) | 147.80 (±18.67) | 2.11 (±0.32) | 8.00 (±0.71) | 12 | - |
| S1A - D | RIM$^{-/-}$ Ib only | 6 | *w;+;$rim^{RS1}$,Is-GAL4/ $rim^{RS1}$,UAS-BoNT-C* | 0.76 (±0.04) | 99.68 (±7.23) | 132.25 (±8.74) | 2.60 (±0.29) | 8.20 (±0.65) | 8 | 0.9986 (ns), 0.9717 (ns), 0.7219 (ns) |
| S1A - D | WT Is only | 6 | *w;+;Ib-GAL4/UAS-BoNT-C* | 1.05 (±0.08) | 156.50 (±7.26) | 156.41 (±10.91) | 3.40 (±0.23) | 7.75 (±0.98) | 9 | - |

| | | | | | | | | | | |
|---|---|---|---|---|---|---|---|---|---|---|
| S1A - D | RIM$^{-/-}$ Is only | 6 | *w;+;rim$^{RS1}$,Ib-GAL4/rim$^{RS1}$,UAS-BoNT-C* | 0.95 (±0.05) | 100.35 (±6.36) | 107.81 (±7.46) | 1.41 (±0.21) | 7.22 (±0.88) | 9 | 0.7486 (ns), 0.0001 (***), 0.0071 (**) |

| Figure | Label | [$Ca^{2+}$] (mM) | Genotype | Failure rate (%) | $R_{input}$ (MΩ) | n | WT vs. *rim* at each condition by unpaired two-tailed Welch's t-test on arcsine-√-transformed data, Holm-Šidák corrected. P value (significance): failure rate. |
|---|---|---|---|---|---|---|---|
| *Additional data* | wild type (Ib+Is) | 0.1 | *w$^{1118}$* | 50.61 (±4.37) | 9.67 (±0.46) | 33 | - |
| *Additional data* | RIM$^{-/-}$ (Ib+Is) | 0.1 | *w;+;rim$^{RS1}$* | 75.53 (±4.32) | 8.42 (±0.50) | 19 | 0.0011 (**) |
| S1E - F | WT Ib only | 0.1 | *w;+;Is-Gal4/UAS-BoNT-C* | 83.25 (±3.84) | 8.25 (±0.49) | 20 | - |
| S1E - F | RIM$^{-/-}$ Ib only | 0.1 | *w;+;rim$^{RS1}$,Is-GAL4/ rim$^{RS1}$,UAS-BoNT-C* | 76.09 (±4.37) | 8.43 (±0.63) | 23 | 0.2843 (ns) |
| S1E - F | WT Is only | 0.1 | *w;+;Ib-GAL4/UAS-BoNT-C* | 55.00 (±6.13) | 9.00 (±0.59) | 25 | - |
| S1E - F | RIM$^{-/-}$ Is only | 0.1 | *w;+;rim$^{RS1}$,Ib-GAL4/rim$^{RS1}$,UAS-BoNT-C* | 72.89 (±4.40) | 7.89 (±0.71) | 19 | 0.0447 (*) |

| Figure | Label | [$Ca^{2+}$] (mM) | Genotype | %EPSC2/EPSC1 | $R_{input}$ (MΩ) | n | WT vs. *rim* at each condition by unpaired two-tailed Welch's t-test on log-transformed PPR, Holm-Šidák corrected. P value (significance): EPSC2/EPSC1. |
|---|---|---|---|---|---|---|---|
| *Additional data* | wild type (Ib+Is) | 0.4 | *w$^{1118}$* | 159.78 (±7.66) | 9.40 (±0.92) | 15 | - |
| *Additional data* | RIM$^{-/-}$ (Ib+Is) | 0.4 | *w;+;rim$^{RS1}$* | 209.95 (±23.48) | 9.06 (±0.94) | 16 | 0.0906 (ns) |
| S1G - H | WT Ib only | 0.4 | *w;+;Is-Gal4/UAS-BoNT-C* | 214.10 (±8.39) | 8.00 (±0.53) | 16 | - |
| S1G - H | RIM$^{-/-}$ Ib only | 0.4 | *w;+;rim$^{RS1}$,Is-GAL4/ rim$^{RS1}$,UAS-BoNT-C* | 196.40 (±13.88) | 6.53 (±0.59) | 15 | 0.1832 (ns) |
| S1G - H | WT Is only | 0.4 | *w;+;Ib-GAL4/UAS-BoNT-C* | 145.30 (±8.58) | 7.67 (±0.48) | 18 | - |
| S1G - H | RIM$^{-/-}$ Is only | 0.4 | *w;+;rim$^{RS1}$,Ib-GAL4/rim$^{RS1}$,UAS-BoNT-C* | 207.90 (±20.21) | 6.94 (±0.59) | 17 | 0.0064 (**) |

| Figure | Label | Genotype | RIM mean intensity (% Ib) | BRP mean intensity (% Ib) | RBP mean intensity (% Ib) | Cac mean intensity (% Ib) | Unc13A mean intensity (% Ib) | Unc13B mean intensity (% Ib) | n | Is vs. Ib by unpaired two-tailed Welch's t-test (log-transformed where required) or Mann-Whitney U test where non-normal. P value (significance): RIM, BRP, RBP / Cac, Unc13A, Unc13B. |
|---|---|---|---|---|---|---|---|---|---|---|
| 2A - B | Is | *w;+;rim$^{ALFA}$* | 124.5 (±4.73) | 86.81 (±2.40) | 79.40 (±2.16) | - | - | - | 40 | <0.0001 (****), 0.0018 (**), <0.0001 (****) |
| 2A, C | Is | *cac$^{sfGFP-N}$;+;+* | -- | -- | -- | 93.73 (±2.95) | 110.60 (±6.08) | 98.95 (±2.03) | 58 (Cac), 40 (Unc13A), 48 (Unc13B) | 0.0766 (ns), 0.8715 (ns), 0.6424 (ns) |

| Figure | Label | Genotype | RIM puncta/BRP ring | n | Is vs. Ib by Mann-Whitney U test. P value (significance): RIM puncta number. |
|---|---|---|---|---|---|
| 2D - E | Ib | *w;+;rim$^{ALFA}$* | 2.38 (±0.11) | 119 | - |
| 2D - E | Is | *w;+;rim$^{ALFA}$* | 2.06 (±0.17) | 47 | 0.0935 (ns) |

| Figure | Label | Genotype | Cac-RIM distance (nm) | n | Is vs. Ib by Mann-Whitney U test. P value (significance): Cac-RIM distance. |
|---|---|---|---|---|---|
| 2F - G | Ib | *cac$^{sfGFP-N}$;+;rim$^{ALFA}$* | 79.35 (±4.30) | 31 | - |
| 2F - G | Is | *cac$^{sfGFP-N}$;+;rim$^{ALFA}$* | 53.91 (±3.22) | 46 | <0.0001 (****) |

| Figure | Label | [$Ca^{2+}$] (mM) | Genotype | BRP density (#/μm$^2$) | mEPSP amplitude (mV) | EPSP (mV) | QC | mEPSP frequency (Hz) | $R_{input}$ (MΩ) | Resting potential (mV) | n (BRP density, electrophysiology) | WT vs. *rim* allele by unpaired two-tailed Welch's t-test (log-transformed where required), Šidák corrected. P value (significance): BRP density, mEPSP, EPSP, QC. |
|---|---|---|---|---|---|---|---|---|---|---|---|---|
| S2C - F | WT | 0.4 | *w$^{1118}$* | 0.82 (±0.05) | 1.03 (±0.05) | 38.80 (±1.79) | 39.21 (±2.81) | 4.22 (±0.52) | 7.42 (±0.51) | -70.25 (±1.51) | 9, 19 | - |
| S2C - F | *rim$^{RS1}$* | 0.4 | *w;+;rim$^{RS1}$* | 0.85 (±0.05) | 1.07 (±0.03) | 18.23 (±1.72) | 17.22 (±1.62) | 2.66 (±0.64) | 6.43 (±0.63) | -68.00 (±1.57) | 7, 14 | 0.9332 (ns), 0.9110 (ns), <0.0001 (****),<0.0001 (****) |
| S2C - F | *rim$^{RS2}$* | 0.4 | *w;+;rim$^{RS2}$* | 0.80 (±0.04) | 0.99 (±0.05) | 17.79 (±1.76) | 19.05 (± 2.67) | 3.45 (±0.32) | 8.20 (±0.58) | -67.85 (±1.12) | 8, 20 | 0.9523 (ns), 0.8686 (ns), <0.0001 (****),<0.0001 (****) |

| Figure | Label | [$Ca^{2+}$] (mM) | Genotype | PhTx | Bouton number | mEPSP amplitude (mV) | EPSP (mV) | QC | mEPSP frequency (Hz) | $R_{input}$ (MΩ) | n | Baseline: WT vs. *rim$^{ALFA}$* by unpaired two-tailed Welch's t-test — P value: bouton number, mEPSP, EPSP, QC. +PhTx: genotype × PhTx interaction assessed by two-way ANOVA; +PhTx vs. baseline compared within each genotype by unpaired two-tailed Welch's t-test (log-transformed where required), Šidák corrected — P value: mEPSP, EPSP, QC. |
|---|---|---|---|---|---|---|---|---|---|---|---|---|
| S2H - K | WT | 0.4 | *w$^{1118}$* | - | 96.70 (±5.25) | 1.06 (±0.02) | 34.40 (±2.20) | 32.71 (± 2.19) | 2.26 (±0.26) | 5.72 (±0.51) | 11 | - |
| S2H - K | Rim$^{ALFA}$ | 0.4 | *w;+;rim$^{ALFA}$* | - | 93.90 (±3.87) | 0.90 (±0.04) | 30.18 (±1.73) | 34.25 (±2.40) | 3.45 (±0.29) | 6.07 (±0.57) | 14 | 0.6734 (ns), 0.0012 (**), 0.1472 (ns), 0.6395 (ns) |
| S2H - K | WT | 0.4 | *w$^{1118}$* | + | -- | 0.54 (±0.02) | 31.61 (±2.53) | 58.66 (± 4.01) | 2.95 (±0.15) | 5.00 (±0) | 9 | <0.0001 (****), 0.6122 (ns), 0.0004 (***) |
| S2H - K | Rim$^{ALFA}$ | 0.4 | *w;+;rim$^{ALFA}$* | + | -- | 0.48 (±0.02) | 28.82 (±1.22) | 60.39 (±3.00) | 3.34 (±0.11) | 5.00 (±0) | 9 | <0.0001 (****), 0.8863 (ns), <0.0001 (****) |

| Figure | Label | Genotype | BRP mean intensity (% WT) | RBP mean intensity (% WT) | Cac mean intensity (% WT) | Unc13A mean intensity (% WT) | Unc13B mean intensity (% WT) | n | WT vs. *rim* at each input (Ib, Is) by unpaired two-tailed Welch's t-test (log-transformed where required) or Mann-Whitney U test where non-normal. P value (significance): BRP, RBP, Cac, Unc13A, Unc13B. |
|---|---|---|---|---|---|---|---|---|---|
| 3A - B | Ib | *cac*$^{sfGFP-N}$;+;*rim*$^{RS1}$ | 105.7 (±2.45) | 101.2 (±2.05) | 94.43 (±1.57) | 97.45 (±6.07) | 99.42 (±1.93) | 92 (BRP/RBP/Cac), 75 (Unc13A/Unc13B) | 0.1236 (ns), 0.5336 (ns), 0.0256 (*), 0.9106 (ns), 0.7347 (ns) |
| 3E - F | Is | *cac*$^{sfGFP-N}$;+;*rim*$^{RS1}$ | 103.8 (±3.04) | 100.9 (±2.49) | 89.39 (±1.89) | 73.50 (±5.04) | 88.52 (±2.82) | 81 (BRP/RBP/Cac), 59 (Unc13A, Unc13B) | 0.9861 (ns), 0.9802 (ns), 0.0002 (***), 0.0064 (**), 0.0003 (***) |

| Figure | Label | Genotype | Cac-Unc13A distance (nm) | n | WT vs. *rim* at each input (Ib, Is) by Mann-Whitney U test. P value (significance): Cac-Unc13A distance. |
|---|---|---|---|---|---|
| 3C - D | WT Ib | *cac*$^{sfGFP-N}$;+;+ | 75.29 (±3.86) | 34 | - |
| 3G - H | RIM$^{-/-}$ Ib | *cac*$^{sfGFP-N}$;+;*rim*$^{RS1}$ | 85.95 (±4.16) | 37 | 0.0460 (*) |
| 3C - D | WT Is | *cac*$^{sfGFP-N}$;+;+ | 70.18 (±3.28) | 55 | - |
| 3G - H | RIM$^{-/-}$ Is | *cac*$^{sfGFP-N}$;+;*rim*$^{RS1}$ | 80.41 (±3.57) | 49 | 0.0432 (*) |

| Figure | Label | Genotype | Cac-Unc13B distance (nm) | n | WT vs. *rim* at each input (Ib, Is) by Mann-Whitney U test. P value (significance): Cac-Unc13B distance. |
|---|---|---|---|---|---|
| *Additional data* | WT Ib | *cac*$^{sfGFP-N}$;+;+ | 124.1 (±4.29) | 44 | - |
| *Additional data* | RIM$^{-/-}$ Ib | *cac*$^{sfGFP-N}$;+;*rim*$^{RS1}$ | 119.5 (±5.39) | 41 | 0.6122 (ns) |
| *Additional data* | WT Is | *cac*$^{sfGFP-N}$;+;+ | 114.1 (±3.28) | 44 | - |
| *Additional data* | RIM$^{-/-}$ Is | *cac*$^{sfGFP-N}$;+;*rim*$^{RS1}$ | 122.9 (±6.52) | 35 | 0.3431 (ns) |

| Figure | Label | [Ca2+] (mM) | Genotype | PhTx | mEPSP amplitude (mV) | EPSP amplitude (mV)/EPSC (nA) | QC | mEPSP frequency (Hz) | $R_{input}$ (MΩ) | Resting potential (mV) | % change in mEPSP amplitude | % change in QC | n | Genotype × treatment interaction assessed by two-way ANOVA; PHP condition vs. baseline compared within each genotype by unpaired two-tailed Welch's t-test (log-transformed where required) or Mann-Whitney U test where non-normal, Šidák corrected. P value (significance): mEPSP, EPSP, QC. |
|---|---|---|---|---|---|---|---|---|---|---|---|---|---|---|
| 4A - B | *IIA* | 0.4 | *w;GluRIIA$^{PV3}$;+* | - | 0.61 (±0.02) | 36.69 (±2.64) | 59.67 (±3.67) | 2.99 (±0.50) | 9.64 (±0.91) | -67.86 (±1.86) | 59.26(±1.45) | 152.19 (±9.91) | 11 | <0.0001 (****), 0.5810 (ns), 0.0002 (***) |
| 4A - B | *IIA* RIM$^{-/-}$ | 0.4 | *w;GluRIIA$^{PV3}$;rim$^{RS1}$* | - | 0.58 (±0.04) | 16.24 (±1.46) | 29.64 (±3.96) | 2.96 (±0.53) | 10.22 (±1.13) | -65.07 (±0.77) | 54.66 (±3.92) | 172.07 (±22.96) | 9 | <0.0001 (****), 0.6249 (ns), 0.0292 (*) |
| 4C - D | *IIA* Ib | 0.4 | w;*GluRIIA$^{PV3}$;Is-GAL4/UAS-BoNT-C* | - | 0.49 (±0.03) | 13.18 (±1.36) | 27.32 (±3.35) | 3.10 (±0.53) | 7.75 (±1.13) | -65.10 (±1.98) | 61.03 (±3.77) | 215.43 (±26.43) | 8 | <0.0001 (****), 0.1455 (ns), 0.0003 (***) |
| 4C - D | *IIA* RIM$^{-/-}$ Ib | 0.4 | *w; GluRIIA$^{PV3}$;rim$^{RS1}$,Is-GAL4/rim$^{RS1}$,UAS-BoNT-C* | - | 0.42 (±0.02) | 12.30 (±2.09) | 30.70 (±6.03) | 4.47 (±0.83) | 7.73 (±0.71) | -66.63 (±1.62) | 51.75 (±2.48) | 237.53 (±46.62) | 11 | <0.0001 (****), 0.5610 (ns), 0.0034 (**) |
| 4E - F | WT | 0.4 | *w$^{1118}$* | + | 0.53 (±0.02) | 33.86 (±2.43) | 64.60 (±4.34) | 1.57 (±0.23) | 8.69 (±0.94) | -67.40 (±2.39) | 51.49 (±2.39) | 163.92 (±11.08) | 13 | <0.0001 (****), 0.2164 (ns), <0.0001 (****) |
| 4E - F | RIM$^{-/-}$ | 0.4 | *w;+;rim$^{RS1}$* | + | 0.60 (±0.01) | 12.83 (±1.10) | 21.34 (±1.86) | 2.40 (±0.18) | 9.05 (±0.72) | -70.34 (±1.44) | 56.70 (±1.25) | 123.88 (±10.80) | 19 | <0.0001 (****), 0.0283 (*), 0.2002 (ns) |
| 4G - H | WT Is | 0.4 | *w;+;Ib-GAL4/UAS-BoNT-C* | + | 0.59 (±0.02) | 32.86 (±1.89) | 56.47 (±3.36) | 3.45 (±0.34) | 7.53 (±0.68) | -67.31 (±1.19) | 49.60 (±1.74) | 248.75 (±14.80) | 15 | <0.0001 (****), 0.0149 (*), <0.0001 (****) |
| 4G - H | RIM$^{-/-}$ Is | 0.4 | *w;+;rim$^{RS1}$,Ib-GAL4/rim$^{RS1}$,UAS-BoNT-C* | + | 0.57 (±0.03) | 5.17 (±0.50) | 9.37 (±1.14) | 2.40 (±0.50) | 8.82 (±0.96) | -66.81 (±1.37) | 49.07 (±2.16) | 97.58 (±11.85) | 11 | <0.0001 (****), 0.0073 (**), 0.8895 (ns) |

| Figure | Label | [$Ca^{2+}$] (mM) | Genotype | PhTx | Cumulative EPSC (nA) | Estimated RRP size | Slope of linear fit | $R_{input}$ (MΩ) | n | WT vs. *rim* at baseline (Ib+Is, Ib, Is) by unpaired two-tailed Welch's t-test (log-transformed where required) or Mann-Whitney U test; ±PhTx vs. baseline at MN-Is by two-way ANOVA (genotype × PhTx), Šidák-corrected multiple comparisons. P value (significance): Slope, Cum. EPSC, RRP size. |
|---|---|---|---|---|---|---|---|---|---|---|
| *Additional data* | wild type (Ib+Is) | 3 | *w$^{1118}$* | - | 963.72 (±80.81) | 946.98 (±81.52) | 45.59 (±3.39) | 8.18 (±0.69) | 11 | - |
| *Additional data* | RIM$^{-/-}$ (Ib+Is) | 3 | *w;+;rim$^{RS1}$* | - | 597.89 (±47.69) | 639.75 (±60.21) | 43.02 (±4.10) | 7.92 (±0.68) | 13 | 0.4662(ns), 0.0012 (**), 0.0041 (**) |
| *Additional data* | WT Ib only | 3 | *w;+;Is-Gal4/UAS-BoNT-C* | - | 344.3 (±60.80) | 413.9 (±66.8) | 43.78 (±3.27) | 9.29 (±0.87) | 12 | - |
| *Additional data* | RIM$^{-/-}$ Ib only | 3 | *w;+;rim$^{RS1}$,Is-GAL4/ rim$^{RS1}$,UAS-BoNT-C* | - | 326.1 (±39.40) | 424.6 (±81.30) | 39.24 (±2.66) | 7.56 (±0.84) | 9 | 0.2942 (ns), 0.8041 (ns), 0.8959 (ns) |
| 5A - C | WT Is only | 3 | *w;+;Ib-GAL4/UAS-BoNT-C* | - | 686.2 (±64.02) | 562.2 (±47.46) | 48.33 (±4.90) | 9.00 (±0.56) | 14 | - |
| 5A - C | RIM$^{-/-}$ Is only | 3 | *w;+;rim$^{RS1}$,Ib-GAL4/rim$^{RS1}$,UAS-BoNT-C* | - | 338.4 (±39.68) | 310.9 (±35.89) | 21.28 (±3.94) | 7.91 (±0.76) | 11 | <0.0001 (****), 0.0001 (***), 0.0003 (***) |
| 5A - C | WT Is only | 3 | *w;+;Ib-GAL4/UAS-BoNT-C* | + | 710.0 (±74.34) | 1324.5 (±154.0) | 35.29 (±4.05) | 7.54 (±0.69) | 13 | 0.1505 (ns), 0.9442 (ns), <0.0001 (****) |

| | | | | | | | | | | |
|---|---|---|---|---|---|---|---|---|---|---|
| 5A - C | RIM$^{-/-}$ Is only | 3 | *w;+;rim$^{RS1}$,Ib-GAL4/rim$^{RS1}$,UAS-BoNT-C* | + | 265.0 (±35.78) | 493.8 (±68.07) | 18.69 (±3.73) | 8.13 (±0.87) | 16 | 0.4761 (ns), 0.5954 (ns), 0.0952 (ns) |
| *Additional data* | WT Is only | 1.5 | *w;+;Ib-GAL4/UAS-BoNT-C* | - | 267.1 (±41.2) | 233.8 (±27.2) | 39.90 (±3.32) | 5.57 (±0.37) | 7 | - |
| *Additional data* | RIM$^{-/-}$ Is only | 1.5 | *w;+;rim$^{RS1}$,Ib-GAL4/rim$^{RS1}$,UAS-BoNT-C* | - | 325.2 (±62.4) | 242.0 (±45.7) | 21.07 (±2.63) | 8.11 (±1.16) | 9 | 0.00015 (***), 0.8000 (ns), 0.9996 (ns) |
| *Additional data* | WT Is only | 1.5 | *w;+;Ib-GAL4/UAS-BoNT-C* | + | 395.3 (±51.4) | 610.4 (±84.6) | 33.70 (±2.95) | 10.44 (±0.88) | 9 | 0.344 (ns), 0.217 (ns), 0.00039 (***) |
| *Additional data* | RIM$^{-/-}$ Is only | 1.5 | *w;+;rim$^{RS1}$,Ib-GAL4/rim$^{RS1}$,UAS-BoNT-C* | + | 155.6 (±26.4) | 296.5 (±48.1) | 18.51 (±2.01) | 8.80 (±0.70) | 10 | 0.866 (ns), 0.038 (*), 0.870 (ns) |

| **Figure** | **Label** | **PhTx** | **Genotype** | **BRP mean intensity (% baseline)** | **RIM mean intensity (% baseline)** | **n** | **Is + PhTx vs. baseline by unpaired two-tailed Welch's t-test (log-transformed where required). P value (significance): BRP, RIM-ALFA.** |
|---|---|---|---|---|---|---|---|
| 6A - B | Is + PhTx | + | *w;+;rim$^{ALFA}$* | 117.6 (±2.19) | 126.9 (±2.84) | 47 | <0.0001 (****), <0.0001 (****) |

| **Figure** | **Label** | **PhTx** | **Genotype** | **RIM puncta/BRP ring** | **Cac-RIM distance (nm)** | **Cac area (nm$^2$)** | **n** | **Is + PhTx vs. baseline by Mann-Whitney U test — P value: RIM puncta, Cac-RIM distance. Cac area: genotype × PhTx interaction assessed by two-way ANOVA; Is + PhTx vs. baseline compared within each genotype by unpaired two-tailed Welch's t-test (log-transformed), Šidák corrected — P value: Cac area.** |
|---|---|---|---|---|---|---|---|---|
| 6C - D | baseline Is | - | *w;+;rim$^{ALFA}$* | 2.03 (±0.13) | - | - | 76 | - |
| 6C, E | Is + PhTx | + | *w;+;rim$^{ALFA}$* | 2.26 (±0.15) | - | - | 70 | 0.2604 (ns) |
| 6C - D | baseline Is | - | *cac$^{sfGFP-N}$;+;rim$^{ALFA}$* | - | 58.95 (±5.82) | - | 19 | - |
| 6C, E | Is + PhTx | + | *cac$^{sfGFP-N}$;+;rim$^{ALFA}$* | - | 38.82 (±3.77) | - | 51 | 0.0026 (**) |
| 6F - G | WT Is | - | *cac$^{sfGFP-N}$;+;+* | - | - | 24.44 (±0.92) | 84 | - |
| 6F - G | WT Is + PhTx | + | *cac$^{sfGFP-N}$;+;+* | - | - | 20.23 (±0.99) | 78 | 0.0007 (***) |
| 6F - G | RIM$^{-/-}$ Is | - | *cac$^{sfGFP-N}$;+;rim$^{RS1}$* | - | - | 19.97 (±1.18) | 46 | - |
| 6F - G | RIM$^{-/-}$ Is + PhTx | + | *cac$^{sfGFP-N}$;+;rim$^{RS1}$* | - | - | 24.88 (±1.18) | 79 | 0.0112 (*) |

| **Figure** | **Label** | **[Ca$^{2+}$] (mM)** | **Genotype** | **PhTx** | **-EGTA baseline EPSC, nA (n)** | **+EGTA EPSC, nA (n)** | **% of baseline** | **$R_{input}$ (MΩ) (+EGTA recordings)** | **By unpaired two-tailed Welch's t-test (log-transformed where required) or Mann-Whitney U test where non-normal, Holm-Šidák corrected. $P_1$: baseline vs. +EGTA (on +PhTx rows, ±PhTx normalized to +EGTA); $P_2$: WT vs. *rim*. Values are % of baseline release remaining after EGTA-AM.** |
|---|---|---|---|---|---|---|---|---|---|
| *Additional data* | wild type (Ib+Is) | 1.8 | *w$^{1118}$* | - | 184.97 (±10.55) (15) | 123.73 (±6.29) (12) | 66.9 (±3.4) | 10.92 (±0.99) | 0.0001 (****) |
| *Additional data* | RIM$^{-/-}$ (Ib+Is) | 1.8 | *w;+;rim$^{RS1}$* | - | 139.56 (± 4.67) (14) | 73.96 (±10.27) (8) | 53.0 (±7.4) | 8.75 (±0.67) | 0.0005 (***), 0.0630 (ns) |

| | | | | | | | | | |
|---|---|---|---|---|---|---|---|---|---|
| *Additional data* | WT Ib only | 1.8 | *w;+;Is-Gal4/UAS-BoNT-C* | - | 64.67 (±5.06) (12) | 30.78 (±4.13) (8) | 47.6 (±6.4) | 8.75 (±0.67) | 0.0011 (**) |
| *Additional data* | RIM$^{-/-}$ Ib only | 1.8 | *w;+;rim*$^{RS1}$*,Is-GAL4/ rim*$^{RS1}$*,UAS-BoNT-C* | - | 76.41 (±6.64) (8) | 38.67 (±4.34) (13) | 50.6 (±5.7) | 8.62 (±0.59) | 0.0004 (***), 0.860 (ns) |
| 7A - B | WT Is only | 1.8 | *w;+;Ib-GAL4/UAS-BoNT-C* | - | 146.97 (±8.89) (9) | 128.03 (±8.02) (12) | 87.1 (±5.5) | 8.89 (±0.61) | 0.131 (ns) |
| 7A - B | RIM$^{-/-}$ Is only | 1.8 | *w;+;rim*$^{RS1}$*,Ib-GAL4/rim*$^{RS1}$*,UAS-BoNT-C* | - | 86.89 (±9.90) (9) | 46.73 (±2.32) (13) | 53.8 (±2.7) | 7.69 (±0.66) | 0.0034 (**), <0.0001 (****) |
| 7A - B | WT Is only | 1.8 | *w;+;Ib-GAL4/UAS-BoNT-C* | + | -- | 105.79 (±5.77) (8) | 82.63 (±4.50) | 5.38 (±0.71) | 0.037 (*) |
| 7A - B | RIM$^{-/-}$ Is only | 1.8 | *w;+;rim*$^{RS1}$*,Ib-GAL4/rim*$^{RS1}$*,UAS-BoNT-C* | + | -- | 51.67 (±3.29) (7) | 110.56 (±7.03) | 7.86 (±1.01) | 0.243 (ns), 0.0070 (**) |

| **Figure** | **Label** | **Genotype** | **PhTx** | **Cac-Unc13A distance (nm)** | **n** | **WT ± PhTx and *rim* ± PhTx (vs. baseline) by Mann-Whitney U test, Holm-Šidák corrected. P value (significance): Cac-Unc13A distance.** |
|---|---|---|---|---|---|---|
| 7C - D | WT Is | *cac*$^{sfGFP-N}$*;+;+* | - | 70.18 (±3.28) | 55 | - |
| 7C - D | WT Is + PhTx | *cac*$^{sfGFP-N}$*;+;+* | + | 54.74 (±2.86) | 57 | 0.0071 (**) |
| 7C - D | RIM$^{-/-}$ Is | *cac*$^{sfGFP-N}$*;+;rim*$^{RS1}$ | - | 80.41 (±3.57) | 49 | - |
| 7C - D | RIM$^{-/-}$ Is + PhTx | *cac*$^{sfGFP-N}$*;+;rim*$^{RS1}$ | + | 84.00 (±5.03) | 45 | 0.7861 (ns) |

## Table S2: Key resources

| REAGENT/RESOURCE | | SOURCE | IDENTIFIER |
|---|---|---|---|
| **Chemicals, peptides, and recombinant proteins** | | | |
| Philanthotoxin-433 | | Sigma-Aldrich | 276684-27-6 |
| EGTA-AM | | Sigma-Aldrich | 99590-86-0 |
| **Antibodies** | **Dilution** | | |
| Mouse anti-BRP (nc82) | 1:250 | Developmental Studies Hybridoma Bank (DSHB) | AB_2314866 |
| Guinea pig anti-RBP | 1:1000 | (*29*) | |
| Rabbit anti-Unc13A | 1:1000 | (*68*) | |
| Guinea pig anti-Unc13B | 1:500 | (*68*) | |
| Mouse anti-GFP | 1:400 | DSHB | AB_2617422 |
| Rabbit anti-ALFA | 1:500 | NanoTag Biotechnologies | AB_3075998 |
| DyLight 405 conjugated secondary antibodies | 1:400 | Jackson ImmunoResearch Laboratories (Jackson) | 715-475-150<br>711-475-152 |
| Alexa Fluor 488 conjugated secondary antibodies | 1:400 | Jackson | 715-545-150<br>715-545-152 |
| Cy3 conjugated secondary antibodies | 1:400 | Jackson | 706-165-148<br>711-165-152<br>715-165-150 |
| Alexa Fluor 594 conjugated secondary antibodies | 1:400 | Jackson | 715-585-150<br>711-585-152 |
| Alexa Fluor 647 conjugated Goat anti-Horseradish Peroxidase | 1:400 | Jackson | 123-605-021 |
| STAR RED conjugated secondary antibodies | 1:200 | Abberior | STRED-1001<br>STRED-1006 |
| **Drosophila Strains** | | | |
| *rim*$^{RS1}$ | | This study | T11H…N31STOP |
| *rim*$^{RS2}$ | | This study | R13H…N33STOP |
| *pGC(2.1)-U6.3_rim_2xgRNA* | | This study | |
| *rim*$^{ALFA}$ | | This study | |
| UAS-BoNT-C | | (*35*) | |
| *cac*$^{sfGFP-N}$ | | (*47*) | |
| dHb9-Gal4 (Ib-Gal4) | | BDSC | 83004 |
| GMR27E09-Gal4 (Is-Gal4) | | BDSC | 49227 |
| OK319-Gal4 | | (*69*) | |
| OK6-Gal4 | | (*70*) | |
| *GluRIIA*$^{pv3}$ | | (*71*) | |
| *Nos.Cas9-DsREd* | | BDSC | FBti0159183 (BDSC #54591) |
| *w*$^{1118}$ | | BDSC | 5905 |

| Software and Algorithms | | |
|---|---|---|
| NIS Elements software | Nikon | 4.51.01 |
| Huygens Essential | SVI | 22.10 |
| Axon pCLAMP | Molecular Devices | 10.7 |
| MiniAnalysis | Synaptosoft | 6.0.3 |
| GraphPad Prism | GraphPad | 8.0.1 |
| ImageJ (Fiji) | (*72*) | |

## REFERENCES


1. T. Biederer, P. S. Kaeser, T. A. Blanpied, Transcellular nanoalignment of synaptic function. *Neuron* **96**, 680–696 (2017).

2. J. S. Dittman, T. A. Ryan, The control of release probability at nerve terminals. *Nat. Rev. Neurosci.* **20**, 177–186 (2019).

3. Z. Nusser, Creating diverse synapses from the same molecules. *Curr. Opin. Neurobiol.* **51**, 8–15 (2018).

4. M. van Oostrum, E. M. Schuman, Understanding the molecular diversity of synapses. *Nat. Rev. Neurosci.* **26**, 65–81 (2025).

5. J. Emperador-Melero, P. S. Kaeser, Assembly of the presynaptic active zone. *Curr. Opin. Neurobiol.* **63**, 95–103 (2020).

6. V. Haucke, E. Neher, S. J. Sigrist, Protein scaffolds in the coupling of synaptic exocytosis and endocytosis. *Nat. Rev. Neurosci.* **12**, 127–138 (2011).

7. P. S. Kaeser, L. Deng, Y. Wang, I. Dulubova, X. Liu, J. Rizo, T. C. Südhof, RIM proteins tether $Ca^{2+}$ channels to presynaptic active zones via a direct PDZ-domain interaction. *Cell* **144**, 282–295 (2011).

8. T. C. Südhof, The presynaptic active zone. *Neuron* **75**, 11–25 (2012).

9. C. Acuna, X. Liu, T. C. Südhof, How to make an active zone: Unexpected universal functional redundancy between RIMs and RIM-BPs. *Neuron* **91**, 792–807 (2016).

10. M. M. Brockmann, F. Zarebidaki, M. Camacho, M. K. Grauel, T. Trimbuch, T. C. Südhof, C. Rosenmund, A trio of active zone proteins comprised of RIM-BPs, RIMs, and Munc13s governs neurotransmitter release. *Cell Rep.* **32**, 107960 (2020).

11. L. Deng, P. S. Kaeser, W. Xu, T. C. Südhof, RIM proteins activate vesicle priming by reversing autoinhibitory homodimerization of Munc13. *Neuron* **69**, 317–331 (2011).

12. J. Emperador-Melero, J. W. Andersen, S. R. Metzbower, A. D. Levy, P. A. Dharmasri, G. de Nola, T. A. Blanpied, P. S. Kaeser, Distinct active zone protein machineries mediate $Ca^{2+}$ channel clustering and vesicle priming at hippocampal synapses. *Nat. Neurosci.* **27**, 1680–1694 (2024).

13. Y. Han, P. S. Kaeser, T. C. Südhof, R. Schneggenburger, RIM determines $Ca^{2+}$ channel density and vesicle docking at the presynaptic active zone. *Neuron* **69**, 304–316 (2011).

14. S. P. Koushika, J. E. Richmond, G. Hadwiger, R. M. Weimer, E. M. Jorgensen, M. L. Nonet, A post-docking role for active zone protein Rim. *Nat. Neurosci.* **4**, 997–1005 (2001).

15. E. R. Graf, V. Valakh, C. M. Wright, C. Wu, Z. Liu, Y. Q. Zhang, A. DiAntonio, RIM promotes calcium channel accumulation at active zones of the *Drosophila* neuromuscular junction. *J. Neurosci.* **32**, 16586–16596 (2012).

16. S. Schoch, P. E. Castillo, T. Jo, K. Mukherjee, M. Geppert, Y. Wang, F. Schmitz, R. C. Malenka, T. C. Südhof, RIM1alpha forms a protein scaffold for regulating neurotransmitter release at the active zone. *Nature* **415**, 321–326 (2002).

17. A. Banerjee, C. Imig, K. Balakrishnan, L. Kershberg, N. Lipstein, R.-L. Uronen, J. Wang, X. Cai, F. Benseler, J. S. Rhee, B. H. Cooper, C. Liu, S. M. Wojcik, N. Brose, P. S. Kaeser, Molecular and functional architecture of striatal dopamine release sites. *Neuron* **110**, 248–265.e9 (2022).

18. C. Liu, L. Kershberg, J. Wang, S. Schneeberger, P. S. Kaeser, Dopamine secretion is mediated by sparse active zone-like release sites. *Cell* **172**, 706–718.e15 (2018).

19. H. Liu, L. Li, D. Nedelcu, Q. Hall, L. Zhou, W. Wang, Y. Yu, J. M. Kaplan, Z. Hu, Heterodimerization of UNC-13/RIM regulates synaptic vesicle release probability but not priming in *C. elegans*. *eLife* **8**, e40585 (2019).

20. C. M. Persoon, R. I. Hoogstraaten, J. P. Nassal, J. R. T. van Weering, P. S. Kaeser, R. F. Toonen, M. Verhage, The RAB3-RIM pathway is essential for the release of neuromodulators. *Neuron* **104**, 1065–1080.e12 (2019).

21. B. G. Robinson, X. Cai, J. Wang, J. R. Bunzow, J. T. Williams, P. S. Kaeser, RIM is essential for stimulated but not spontaneous somatodendritic dopamine release in the midbrain. *eLife* **8**, e47972 (2019).

22. J. J. Bruckner, H. Zhan, S. J. Gratz, M. Rao, F. Ukken, G. Zilberg, K. M. O'Connor-Giles, Fife organizes synaptic vesicles and calcium channels for high-probability neurotransmitter release. *J. Cell Biol.* **216**, 231–246 (2017).

23. J. J. Bruckner, S. J. Gratz, J. K. Slind, R. R. Geske, A. M. Cummings, S. E. Galindo, L. K. Donohue, K. M. O'Connor-Giles, Fife, a *Drosophila* Piccolo-RIM homolog, promotes active zone organization and neurotransmitter release. *J. Neurosci.* **32**, 17048–17058 (2012).

24. K. S. Y. Liu, M. Siebert, S. Mertel, E. Knoche, S. Wegener, C. Wichmann, T. Matkovic, K. Muhammad, H. Depner, C. Mettke, J. Bückers, S. W. Hell, M. Müller, G. W. Davis, D. Schmitz, S. J. Sigrist, RIM-binding protein, a central part of the active zone, is essential for neurotransmitter release. *Science* **334**, 1565–1569 (2011).

25. R. J. Kittel, C. Wichmann, T. M. Rasse, W. Fouquet, M. Schmidt, A. Schmid, D. A. Wagh, C. Pawlu, R. R. Kellner, K. I. Willig, S. W. Hell, E. Buchner, M. Heckmann, S. J. Sigrist, Bruchpilot promotes active zone assembly, $Ca^{2+}$ channel clustering, and vesicle release. *Science* **312**, 1051–1054 (2006).

26. M. Müller, K. S. Y. Liu, S. J. Sigrist, G. W. Davis, RIM controls homeostatic plasticity through modulation of the readily-releasable vesicle pool. *J. Neurosci.* **32**, 16574–16585 (2012).

27. N. A. Aponte-Santiago, J. T. Littleton, Synaptic properties and plasticity mechanisms of invertebrate tonic and phasic neurons. *Front. Physiol.* **11**, 611982 (2020).

28. C. Chien, K. He, S. Perry, E. Tchitchkan, Y. Han, X. Li, D. Dickman, Distinct input-specific mechanisms enable presynaptic homeostatic plasticity. *Sci. Adv.* **11**, eadr0262 (2025).

29. K. He, Y. Han, X. Li, R. X. Hernandez, D. V. Riboul, T. Feghhi, K. A. Justs, O. Mahneva, S. Perry, G. T. Macleod, D. Dickman, Physiologic and nanoscale distinctions define glutamatergic synapses in tonic vs phasic neurons. *J. Neurosci.* **43**, 4598–4611 (2023).

30. K. He, D. Dickman, Building and modifying diverse synaptic properties: Insights from *Drosophila*. *Curr. Opin. Neurobiol.* **92**, 102995 (2025).

31. S. K. Jetti, A. B. Crane, Y. Akbergenova, N. A. Aponte-Santiago, K. L. Cunningham, C. A. Whittaker, J. T. Littleton, Molecular logic of synaptic diversity between *Drosophila* tonic and phasic motoneurons. *Neuron* **111**, 3554–3569.e7 (2023).

32. A. T. Medeiros, S. J. Gratz, A. Delgado, J. T. Ritt, K. M. O'Connor-Giles, Molecular and organizational diversity intersect to generate functional synaptic heterogeneity within and between excitatory neuronal subtypes. *eLife* **12**, RP88412 (2024).

33. Z. Lu, A. K. Chouhan, J. A. Borycz, Z. Lu, A. J. Rossano, K. L. Brain, Y. Zhou, I. A. Meinertzhagen, G. T. Macleod, High-probability neurotransmitter release sites represent an energy-efficient design. *Curr. Biol.* **26**, 2562–2571 (2016).

34. Z. L. Newman, A. Hoagland, K. Aghi, K. Worden, S. L. Levy, J. H. Son, L. P. Lee, E. Y. Isacoff, Input-specific plasticity and homeostasis at the *Drosophila* larval neuromuscular junction. *Neuron* **93**, 1388–1404.e10 (2017).

35. Y. Han, C. Chien, P. Goel, K. He, C. Pinales, C. Buser, D. Dickman, Botulinum neurotoxin accurately separates tonic vs. phasic transmission and reveals heterosynaptic plasticity rules in *Drosophila*. *eLife* **11**, e77924 (2022).

36. A. Mrestani, S. Dannhäuser, M. Pauli, P. Kollmannsberger, M. Hübsch, L. Morris, T. Langenhan, M. Heckmann, M. M. Paul, Nanoscaled RIM clustering at presynaptic active zones revealed by endogenous tagging. *Life Sci. Alliance* **6**, e202302021 (2023).

37. H. Götzke, M. Kilisch, M. Martínez-Carranza, S. Sograte-Idrissi, A. Rajavel, T. Schlichthaerle, N. Engels, R. Jungmann, P. Stenmark, F. Opazo, S. Frey, The ALFA-tag is a highly versatile tool for nanobody-based bioscience applications. *Nat. Commun.* **10**, 4403 (2019).

38. M. A. Böhme, C. Beis, S. Reddy-Alla, E. Reynolds, M. M. Mampell, A. T. Grasskamp, J. Lützkendorf, D. D. Bergeron, J. H. Driller, H. Babikir, F. Göttfert, I. M. Robinson, C. J. O'Kane, S. W. Hell, M. C. Wahl, U. Stelzl, B. Loll, A. M. Walter, S. J. Sigrist, Active zone scaffolds

differentially accumulate Unc13 isoforms to tune $Ca^{2+}$ channel-vesicle coupling. *Nat. Neurosci.* **19**, 1311–1320 (2016).

39. G. W. Davis, M. Müller, Homeostatic control of presynaptic neurotransmitter release. *Annu. Rev. Physiol.* **77**, 251–270 (2015).

40. C. A. Frank, M. J. Kennedy, C. P. Goold, K. W. Marek, G. W. Davis, Mechanisms underlying the rapid induction and sustained expression of synaptic homeostasis. *Neuron* **52**, 663–677 (2006).

41. C. A. Frank, T. D. James, M. Müller, Homeostatic control of *Drosophila* neuromuscular junction function. *Synapse* **74**, e22133 (2020).

42. P. Goel, D. Dickman, Synaptic homeostats: Latent plasticity revealed at the *Drosophila* neuromuscular junction. *Cell. Mol. Life Sci.* **78**, 3159–3179 (2021).

43. P. Goel, X. Li, D. Dickman, Estimation of the readily releasable synaptic vesicle pool at the *Drosophila* larval neuromuscular junction. *Bio Protoc.* **9**, e3127 (2019).

44. M. A. Böhme, A. W. McCarthy, A. T. Grasskamp, C. B. Beuschel, P. Goel, M. Jusyte, D. Laber, S. Huang, U. Rey, A. G. Petzoldt, M. Lehmann, F. Göttfert, P. Haghighi, S. W. Hell, D. Owald, D. Dickman, S. J. Sigrist, A. M. Walter, Rapid active zone remodeling consolidates presynaptic potentiation. *Nat. Commun.* **10**, 1085 (2019).

45. P. Goel, D. Dufour Bergeron, M. A. Böhme, L. Nunnelly, M. Lehmann, C. Buser, A. M. Walter, S. J. Sigrist, D. Dickman, Homeostatic scaling of active zone scaffolds maintains global synaptic strength. *J. Cell Biol.* **218**, 1706–1724 (2019).

46. P. Goel, X. Li, D. Dickman, Disparate postsynaptic induction mechanisms ultimately converge to drive the retrograde enhancement of presynaptic efficacy. *Cell Rep.* **21**, 2339–2347 (2017).

47. S. J. Gratz, P. Goel, J. J. Bruckner, R. X. Hernandez, K. Khateeb, G. T. Macleod, D. Dickman, K. M. O'Connor-Giles, Endogenous tagging reveals differential regulation of $Ca^{2+}$ channels at

single active zones during presynaptic homeostatic potentiation and depression. *J. Neurosci.* **39**, 2416–2429 (2019).

48. A. Weyhersmüller, S. Hallermann, N. Wagner, J. Eilers, Rapid active zone remodeling during synaptic plasticity. *J. Neurosci.* **31**, 6041–6052 (2011).

49. T. Ghelani, M. Escher, U. Thomas, K. Esch, J. Lützkendorf, H. Depner, M. Maglione, P. Parutto, S. Gratz, T. Matkovic-Rachid, S. Ryglewski, A. M. Walter, D. Holcman, K. O'Connor Giles, M. Heine, S. J. Sigrist, Interactive nanocluster compaction of the ELKS scaffold and Cacophony $Ca^{2+}$ channels drives sustained active zone potentiation. *Sci. Adv.* **9**, eade7804 (2023).

50. P. S. Kaeser, W. G. Regehr, Molecular mechanisms for synchronous, asynchronous, and spontaneous neurotransmitter release. *Annu. Rev. Physiol.* **76**, 333–363 (2014).

51. C. J. Meinrenken, J. G. G. Borst, B. Sakmann, Calcium-secretion coupling at Calyx of Held governed by nonuniform channel–vesicle topography. *J. Neurosci.* **22**, 1648–1667 (2002).

52. T. H. Nguyen, R. Vicidomini, S. D. Choudhury, T. H. Han, D. Maric, T. Brody, M. Serpe, scRNA-seq data from the larval *Drosophila* ventral cord provides a resource for studying motor systems function and development. *Dev. Cell* **59**, 1210–1230.e9 (2024).

53. Z. L. Newman, D. Bakshinskaya, R. Schultz, S. J. Kenny, S. Moon, K. Aghi, C. Stanley, N. Marnani, R. Li, J. Bleier, K. Xu, E. Y. Isacoff, Determinants of synapse diversity revealed by super-resolution quantal transmission and active zone imaging. *Nat. Commun.* **13**, 229 (2022).

54. P. S. Kaeser, L. Deng, M. Fan, T. C. Südhof, RIM genes differentially contribute to organizing presynaptic release sites. *Proc. Natl. Acad. Sci. U.S.A.* **109**, 11830–11835 (2012).

55. A. Mrestani, M. Pauli, P. Kollmannsberger, F. Repp, R. J. Kittel, J. Eilers, S. Doose, M. Sauer, A.-L. Sirén, M. Heckmann, M. M. Paul, Active zone compaction correlates with presynaptic homeostatic potentiation. *Cell Rep.* **37**, 109770 (2021).

56. X. Chen, X. Wu, H. Wu, M. Zhang, Phase separation at the synapse. *Nat. Neurosci.* **23**, 301–310 (2020).

57. X. Wu, Q. Cai, Z. Shen, X. Chen, M. Zeng, S. Du, M. Zhang, RIM and RIM-BP form presynaptic active-zone-like condensates via phase separation. *Mol. Cell* **73**, 971–984.e5 (2019).

58. A. G. Nair, P. Muttathukunnel, M. Müller, Distinct molecular pathways govern presynaptic homeostatic plasticity. *Cell Rep.* **37**, 110105 (2021).

59. P. S. Kaeser, H.-B. Kwon, C. Q. Chiu, L. Deng, P. E. Castillo, T. C. Südhof, RIM1α and RIM1β are synthesized from distinct promoters of the RIM1 gene to mediate differential but overlapping synaptic functions. *J. Neurosci.* **28**, 13435–13447 (2008).

60. P. E. Castillo, S. Schoch, F. Schmitz, T. C. Südhof, R. C. Malenka, RIM1α is required for presynaptic long-term potentiation. *Nature* **415**, 327–330 (2002).

61. X. Wu, Z. Shen, M. Zhang, Phase separation-mediated compartmentalization underlies synapse formation and plasticity. *Annu. Rev. Neurosci.* **48**, 149–168 (2025).

62. S. Zhu, Z. Shen, X. Wu, M. Zhang, Phase separation in the multi-compartment organization of synapses. *Curr. Opin. Neurobiol.* **90**, 102975 (2025).

63. B. Kiragasi, J. Wondolowski, Y. Li, D. K. Dickman, A presynaptic glutamate receptor subunit confers robustness to neurotransmission and homeostatic potentiation. *Cell Rep.* **19**, 2694–2706 (2017).

64. K. Kikuma, X. Li, D. Kim, D. Sutter, D. K. Dickman, Extended synaptotagmin localizes to presynaptic ER and promotes neurotransmission and synaptic growth in *Drosophila*. *Genetics* **207**, 993–1006 (2017).

65. B. Kiragasi, P. Goel, S. Perry, Y. Han, X. Li, D. Dickman, The auxiliary glutamate receptor subunit dSol-1 promotes presynaptic neurotransmitter release and homeostatic potentiation. *Proc. Natl. Acad. Sci. U.S.A.* **117**, 25830–25839 (2020).

66. C. Qiu, S. Perry, C. Chen, J. Chen, J. Zhuang, Y. Han, P. Goel, D. Dickman, Nonionic signaling rapidly remodels postsynaptic DLG to induce retrograde homeostatic plasticity. *Proc. Natl. Acad. Sci. U.S.A.* **122**, e2502997122 (2025).

67. J. O. Wobbrock, L. Findlater, D. Gergle, J. J. Higgins, "The aligned rank transform for nonparametric factorial analyses using only ANOVA procedures" in *Proceedings of the SIGCHI Conference on Human Factors in Computing Systems (CHI '11)* (ACM, 2011), pp. 143–146.

68. A. Pooryasin, M. Maglione, M. Schubert, T. Matkovic-Rachid, S. Hasheminasab, U. Pech, A. Fiala, T. Mielke, S. J. Sigrist, Unc13A and Unc13B contribute to the decoding of distinct sensory information in *Drosophila*. *Nat. Commun.* **12**, 1932 (2021).

69. E. S. Beck, G. Gasque, W. L. Imlach, W. Jiao, B. Jiwon Choi, P.-S. Wu, M. L. Kraushar, B. D. McCabe, Regulation of Fasciclin II and synaptic terminal development by the splicing factor beag. *J. Neurosci.* **32**, 7058–7073 (2012).

70. H. Aberle, A. P. Haghighi, R. D. Fetter, B. D. McCabe, T. R. Magalhães, C. S. Goodman, Wishful thinking encodes a BMP type II receptor that regulates synaptic growth in *Drosophila*. *Neuron* **33**, 545–558 (2002).

71. Y. Han, P. Goel, J. Chen, S. Perry, N. Tran, S. Nishimura, M. Sanjani, C. Chien, D. Dickman, Excess glutamate release triggers subunit-specific homeostatic receptor scaling. *Cell Rep.* **42**, 112775 (2023).

72. C. T. Rueden, J. Schindelin, M. C. Hiner, B. E. DeZonia, A. E. Walter, E. T. Arena, K. W. Eliceiri, ImageJ2: ImageJ for the next generation of scientific image data. *BMC Bioinformatics* **18**, 529 (2017).
